# Ex vivo drug response heterogeneity reveals personalized therapeutic strategies for patients with multiple myeloma

**DOI:** 10.1038/s43018-023-00544-9

**Published:** 2023-04-20

**Authors:** Klara Kropivsek, Paul Kachel, Sandra Goetze, Rebekka Wegmann, Yasmin Festl, Yannik Severin, Benjamin D. Hale, Julien Mena, Audrey van Drogen, Nadja Dietliker, Joëlle Tchinda, Bernd Wollscheid, Markus G. Manz, Berend Snijder

**Affiliations:** 1 Institute of Molecular Systems Biology, Department of Biology, ETH Zurich, Zurich, Switzerland; 2 Swiss Institute of Bioinformatics, Lausanne, Switzerland; 3 Department of Medical Oncology and Hematology, University Hospital Zurich and University of Zurich, Zurich, Switzerland; 4 Institute of Translational Medicine, Department of Health Sciences and Technology, ETH Zurich, Zurich, Switzerland; 5 Swiss Multi-Omics Center, PHRT-CPAC, ETH Zurich, Zurich, Switzerland; 6 Pediatric Oncology, Children’s Research Centre, University Children’s Hospital Zurich, Zurich, Switzerland; 7 Comprehensive Cancer Center Zurich (CCCZ), Zurich, Switzerland

**Keywords:** Myeloma, Phenotypic screening, Cancer therapy, Cancer

## Abstract

Multiple myeloma (MM) is a plasma cell malignancy defined by complex genetics and extensive patient heterogeneity. Despite a growing arsenal of approved therapies, MM remains incurable and in need of guidelines to identify effective personalized treatments. Here, we survey the ex vivo drug and immunotherapy sensitivities across 101 bone marrow samples from 70 patients with MM using multiplexed immunofluorescence, automated microscopy and deep-learning-based single-cell phenotyping. Combined with sample-matched genetics, proteotyping and cytokine profiling, we map the molecular regulatory network of drug sensitivity, implicating the DNA repair pathway and EYA3 expression in proteasome inhibitor sensitivity and major histocompatibility complex class II expression in the response to elotuzumab. Globally, ex vivo drug sensitivity associated with bone marrow microenvironmental signatures reflecting treatment stage, clonality and inflammation. Furthermore, ex vivo drug sensitivity significantly stratified clinical treatment responses, including to immunotherapy. Taken together, our study provides molecular and actionable insights into diverse treatment strategies for patients with MM.

## Abstract (web summary)

Snijder and colleagues use ex vivo pharmacoscopy and bone marrow composition profiling in a cohort of patients with multiple myeloma to identify tailored therapeutic sensitivities and stratify the cohort into three microenvironmental PhenoGroups.

## Main

MM is a cancer driven by malignant plasma cells (myeloma cells) in the bone marrow (BM)^1,2^. Patients with MM require treatment to decrease the malignant plasma cell counts, fight the detrimental effects of the disease, improve quality of life and prolong survival. The events that lead to malignant transformation are in part intrinsic to healthy plasma cell function. These include the genetic rearrangements and cellular adaptations required for the production of diverse antibody repertoires and the longevity, supported by the BM niche, required for long-term immunity^3,4^. Aberrant recombination can lead to chromosomal translocations, a central characteristic of MM^5^. Over 90% of translocations affect chromosome 14, specifically the *IGH* locus at *14q32.22*, which is among the most heavily transcribed genes in plasma cells^6^. The resulting fusion products can place partner genes under the control of the *IGH* enhancer, leading, for example, to deregulation of cyclin D expression^7^. Genes involved in B-cell lineage differentiation are frequently mutated in multiple myeloma, including *PRDM1* and interferon regulatory factor 4 (*IRF4*), a key driver of MM^5,8,9^. In all, chromosomal gains, deletions, translocations and gene mutations increase the genetic complexity while enhancing myeloma cell survival and proliferative capacity.

The BM environment contributes to disease progression and treatment response by providing a protective niche and proliferative factors to myeloma cells^10^. Myeloma niche interactions are reflected in altered BM composition of both immune, stromal, osteoclast and osteoblast cells, as well as altered levels of secreted factors^11,12^. Cytokines tumor necrosis factor (TNF)-α and interleukin (IL)-6, for example, are provided by the niche and can induce proliferation of myeloma cells, creating a tumor-supportive microenvironment^13,14^.

Many treatment options exist for MM that together have markedly prolonged overall survival in the last decades. Advances in MM therapy and management came from the introduction of proteasome inhibitors (PIs; for example, bortezomib, carfilzomib and ixazomib) and immunomodulatory agents (IMiDs; for example, thalidomide, lenalidomide and pomalidomide)^15^. These are commonly given as double- or triple-drug combinations with corticosteroids (dexamethasone or prednisone). Deep responses to these therapies correlate with a longer time to relapse and improved overall survival^16^ and are commonly followed by autologous stem cell transplantation in younger and fit patients^17^. Introduction of targeted immunotherapies has further brought clinical benefits to patients with MM, often in combinations with the above-mentioned small compound therapies^18–20^. These immunotherapies include monoclonal antibodies directed against myeloma surface antigens, such as daratumumab targeting CD38 (ref. ^21^) and elotuzumab targeting SLAMF7/CD319 (refs. ^22,23^), as well as BCMA-targeted chimeric antigen receptor (CAR) T cell therapy^24^.

Despite this plethora of approved treatment options, MM is still incurable and most patients eventually relapse and die of the illness^25^. Myeloma cells evade treatment both by innate^26,27^ and treatment-induced mechanisms^28,29^. A well-studied example is the deletion of chromosome arm 17p (del17p), frequently acquired during disease progression. Del17p is associated with mutations in *TP53* and with a more aggressive disease that responds poorly to proteasome inhibition^28,30–32^.

MM has been extensively analyzed by comprehensive molecular profiling (‘omics’) techniques. Several studies used genomic^9,33^, transcriptomic^34^ and proteomic^35–37^ measurements to strengthen the knowledge of disease biology, identify drug targets, predict the risk of progression^38^ and explain acquired resistance mechanisms^26,39^; however, omics measurements have not yet transformed the clinical routine for MM.

Complementary to molecularly guided precision medicine, drug testing directly on patient biopsies is increasingly successfully implemented for personalized treatment selection^40–42^ and analysis of drug sensitivity^43–45^. For MM, several ex vivo drug screening studies have been performed with bulk viability assays informing on possible treatment options, including BCL2 inhibition by venetoclax^46–48^. We have recently reported that image-based ex vivo drug testing (called pharmacoscopy; PCY) recommends treatments that lead to significant progression-free survival improvement for patients suffering from relapsed/refractory hematological malignancies, compared to the patient’s own responses to previous treatment^40,41,49^. This high-content single-cell approach also allows measurement of ex vivo sensitivity to immunotherapies, by quantifying immune cell activation, target cell engagement and target cell killing^50–52^. Based on these observations, we set out to adapt PCY to MM, use it at scale to systematically analyze disease heterogeneity in treatment sensitivity and resistance and evaluate the clinical predictive power of the approach for MM.

## Results

**Fig. 1:**
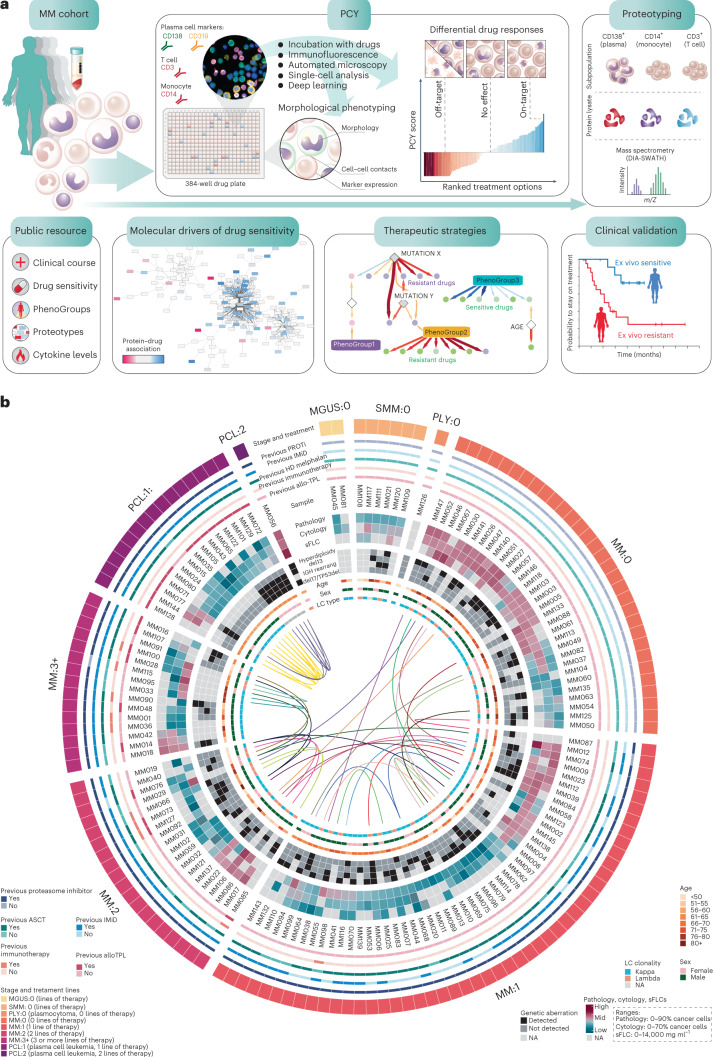
Workflow and cohort details for the integrative functional, molecular and clinical analysis of patients with MM. **a**, Schematic indicating the study workflow and derived results. Data are available as described in the Data Availability section, as well as at https://myelomics.com. **b**, Circos plot representing the multiple myeloma cohort and samples collected during the observational clinical study. A total of 138 patient samples from 89 unique patients are shown, with the follow-up samples from recurring patients connected with the lines in the inner part of the circos plot (unique color per patient). sFLC, serum-free light chain; NA, not available. For further details see legend, Extended Data Fig. 1 and Supplementary Table 1.

**Extended Data Fig. 1:**
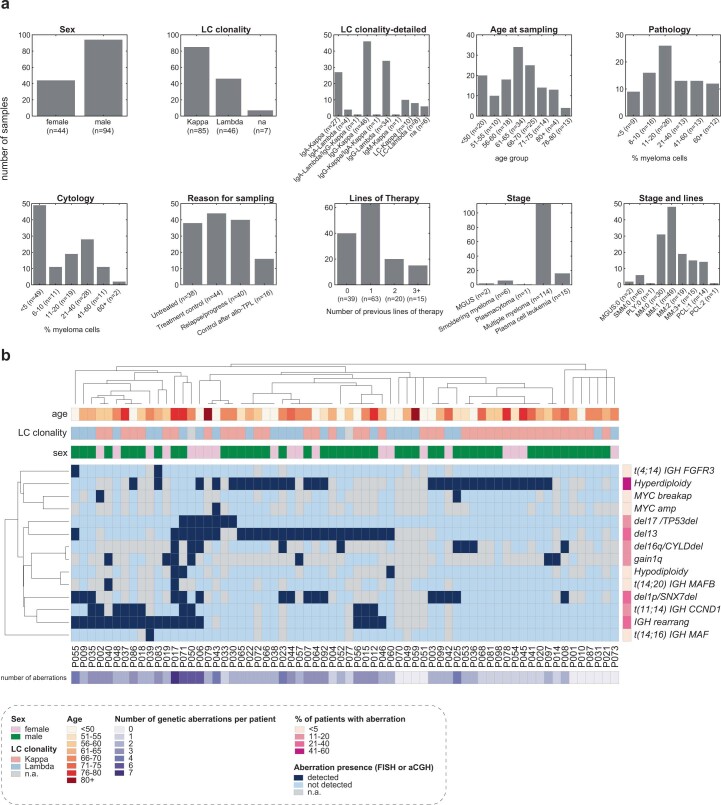
Patient cohort details and genetics. **a**, Histograms of patient clinical features, for 138 patient samples. Ns represent the number of patient samples for which the clinical feature measurement exists. **b**, Heatmap of genetic aberrations, as measured in the clinic at the time of patient’s diagnosis by fluorescence in situ hybridization (FISH) or Microarray-based Comparative Genomic Hybridization (aCGH). Rows correspond to individual mutations, and columns to individual patients (only shown for those that such data was measured; n = 66). Additionally, age, light chain clonality, and sex per patient are shown (see legend). On the right, the % of patients with the mutation detected in the cohort is indicated (see legend). Below the patient labels, the number of detected mutations per patient is indicated (Supplementary Table 1). Source data

MM (or plasma cell myeloma) is characterized by the clonal expansion of myeloma cells (malignant plasma cells) and accumulation of genetic lesions, leading to extensive intra- and inter-patient heterogeneity^5,34,39^. To assess the impact of this heterogeneity on drug responses and identify therapeutic strategies, we combined ex vivo image-based drug screening (PCY) combined with data-independent acquisition (DIA)-sliding window of all theoretical mass spectra (SWATH) proteotyping^53^ (Fig. 1a) of real-time BM aspirates from a clinically representative patient cohort (Fig. 1b; ethical approval number BASEC: 2017-00603). We collected 138 BM samples from 89 unique patients (Supplementary Table 1). Nine samples were additionally collected, but were excluded from the final analysis because of insufficient material or the diagnosis not matching. The patient stages at the time of sampling range from precursor stages monoclonal gammopathy of undetermined significance, smoldering MM (SMM) and untreated MM (MM:0) to samples from patients with MM after three or more previous lines of therapy (MM:3+) and extramedullary disease, plasma cell leukemia (Fig. 1b). Depending on sample availability, we combined PCY testing with proteotyping^53^ of magnetic bead-based enriched CD138^+^ plasma cells, CD14^+^ monocytes and CD3^+^ T cells and integrated these results with the matched clinical data (Fig. 1b and Extended Data Fig. 1a,b).

### Single-cell analysis of complex bone marrow samples

**Fig. 2:**
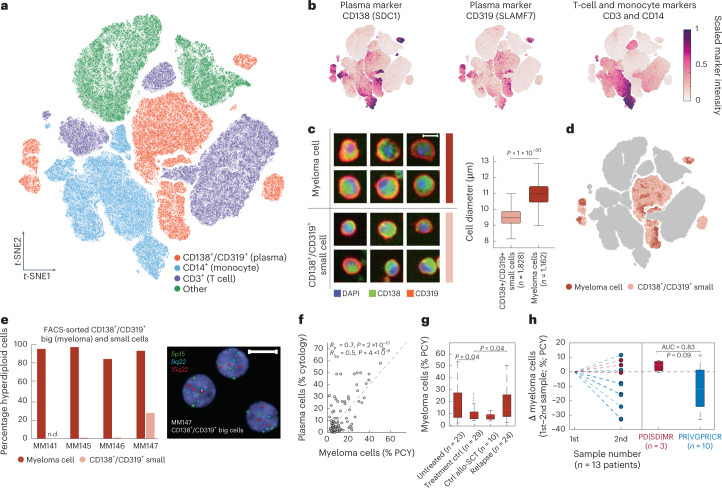
A clinically concordant morphological signature of malignant myeloma cells. **a**, *t*-Stochastic neighbor embedding (*t*-SNE) of the CNN latent space of high-confidence cells colored by CNN class (*n* = 489,753 cells from 97 patient samples). **b**, Marker expression levels per cell projected onto the embedding of **a**. **c**, Example cropped microscopy images showing representative morphologies of myeloma cells (top) and small CD138^+^/CD319^+^ plasma cell-marker-positive cells (bottom). Scale bar, 10 µm. Box-plots of cell diameter of myeloma cells (*n* = 1,828 cells from 55 patient samples) and small plasma cell-marker-positive cells (*n* = 1,162 cells from 55 patient samples) (right). Box-plots indicate the median (horizontal line) and 25% and 75% ranges (box) and whiskers indicate the 1.5 × interquartile range above or below the box. Outliers beyond this range are shown as individual data points. In this case no outliers are present. *P* values from unpaired two-tailed Student’s *t*-test. **d**, Plasma cell class morphology projected onto the embedding of **a**. **e**, DNA-fluorescence in-situ hybridization (FISH) results assessing hyperdiploidy of FACS-sorted plasma cells (CD138^+^ or CD319^+^) that were further subdivided by size (see also Extended Data Fig. 2e). Bar graphs represent 100 cells per class for four patient samples. Example FISH-image of sample MM147 indicating hyperdiploidy for three nuclei (right). Blue indicates 4,6-diamidino-2-phenylindole (DAPI) stain. Scale bar, 10 µm. **f**, Scatter-plot of percentage myeloma cells by PCY compared to evaluation by clinical cytology (*n* = 82 patient samples). Spearman’s rank and Pearson’s correlations and *P* values are indicated. **g**, Box-plot of percentage myeloma cells by PCY stratified by treatment stage (*n* = 86 patient samples). *P* values from multiple pairwise comparison of the group means using Tukey’s honestly significant difference criterion. Data are not adjusted for multiple comparisons. Box-plots as in **c**. **h**, Difference in percentage myeloma cells in longitudinal patient samples, normalized to the first sampling. Red indicates patients with less than PR; blue shows patients with PR or better. Box-plots as in **c**. *P* values from paired two-tailed *t*-test. AUC, area under the receiver operating characteristic curve; PD, progressive disease; SD, stable disease; MR, minimal response; VGPR, very good partial response; CR, complete remission, as defined by the International Myeloma Working Group. Source data

**Extended Data Fig. 2:**
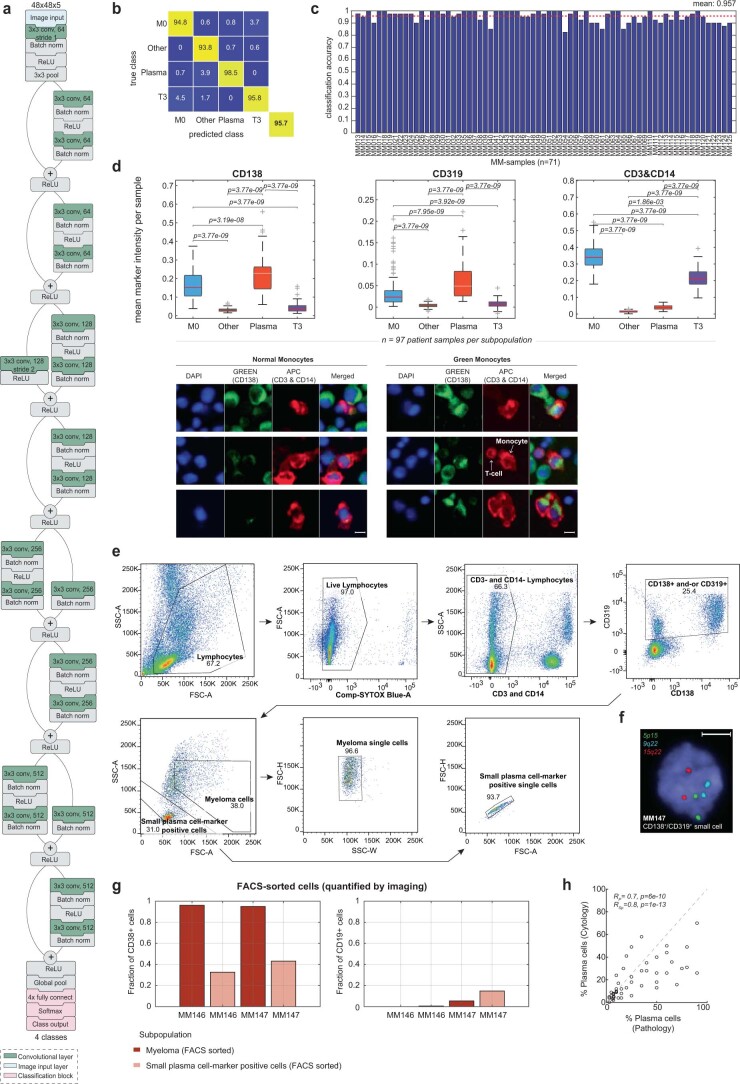
Single-cell image analysis of primary myeloma samples. **a**, Convolutional neural network architecture (ResNet) of the 4-class classifier used in the study to classify each imaged cell into either a monocyte, plasma cell, T cell, or other cell class. **b**, Confusion matrix showing the ResNet classification accuracy on cells from 71 samples used in the training set, calculated on cells that were not part of the CNN training data. Overall classification accuracy across the training samples was 95.7%, with the highest accuracy for the plasma cell class (98.5% accuracy). **c**, Overall classification accuracy per patient sample, calculated on cells that were not part of the CNN training data (n = 71 samples). Red dashed line indicates average accuracy across all samples. **d**, Boxplots of mean marker intensities per sample (n = 97 patient samples) of each respective subpopulation marker (n = 1260 cells on average per subpopulation and marker and sample). Boxplots as in Fig. 2c. P-values calculated by unpaired two-tailed Student’s t-test. **d, below**, Image examples of normal and green monocytes. Scale bar: 10 μm. **e**, Exemplary FACS-gating for sorting of myeloma and small plasma cell-marker-positive cells. First, FSC-A and SSC-A gates are used to select the lymphocytes that are further enriched in viable cells. CD14 and CD3 cells are excluded, and only plasma cell-marker (CD138 and/or CD319) positive cells are kept. Finally, plasma cell-marker-positive cells are separated based on SSC-A and FSC-A gates into big cells (myeloma cells) and small plasma cell-marker-positive cells. Singlets of each subpopulation are chosen and sorted out for further downstream processing. **f**, Example FISH-image of a small plasma cell-marker-positive cell of MM147 sample, showing normal chromosomal copy numbers (diploidy) for the tested probes. Picture is representative of 100 cells analyzed for this sample and class, see quantification in Fig. 2e. Scale bar: 10 µm. **g**, Barplots showing fraction of myeloma (big) and small FACS-sorted CD138^+^/CD319^+^ cells positive by immunofluorescence and imaging for either CD38 (plasma cell marker) or CD19 (B-cell marker) (n = 10,000 cells). **h**, Scatterplot of the percentage of plasma cells infiltrated into the bone marrow measured on matching samples by either clinical pathology (x-axis) or clinical cytology (y-axis) (n = 50 samples). Spearman’s rank and Pearson’s correlations and p-values are reported. Source data

The generated image-based dataset, comprising 729 million imaged BM-residing mononuclear cells (BMNCs), offers a unique phenotypic view on cellular heterogeneity in MM. To quantify this heterogeneity, a convolutional neural network (CNN)^54^ (Extended Data Fig. 2a–c) first classified each imaged BMNC into either CD138^+^/CD319^+^ plasma cell-marker-positive cells, CD3^+^ T cells, CD14^+^ monocytes or an ‘other’ class for all marker-negative cells (Extended Data Fig. 2d). Convolutional neural networks learn latent-space representations that cluster data with similar features, useful for the discovery of subpopulations of cells with similar phenotypic features in single-cell imaging^52^. Within our cohort, such visualization of the latent-space features (activations of the CNN’s last fully connected layer; Fig. 2a) of cells from control conditions revealed considerable phenotypic heterogeneity across the cohort (Fig. 2b). This included abundant bona fide CD14^+^ monocytes with unexpected intracellular green signal (Extended Data Fig. 2d), as well as considerable cell size variability within the plasma cell-marker-positive cell class (Fig. 2c).

### High-throughput phenotypic detection of myeloma cells

Myeloma cells are characterized by large cytoplasms^55^, positive expression of plasma cell markers (including CD38, CD138 and CD319) and cytogenetic aberrations^5^. Therefore, a second neural network classified the subset of big plasma cell-marker-positive cells, which are the putative myeloma cells that form the target population for the ex vivo drug screens (Fig. 2d). We confirmed this myeloma cell identification strategy using molecular and genetic analysis of FACS-sorted CD138^+^/CD319^+^ plasma cell-marker-positive big and small cells (Extended Data Fig. 2e). Indeed, across four validation samples, the majority of big cells were hyperdiploid (Fig. 2e) and additionally expressed the plasma cell marker CD38 (Extended Data Fig. 2g). In contrast, the plasma-marker-positive small cells were nearly completely diploid and only partially CD38-positive and thus not all bona fide plasma cells (Fig. 2e and Extended Data Fig. 2f,g). Neither big nor small plasma cell marker-positive cells were immature plasma or B cells, as both were B-cell marker CD19-negative (Extended Data Fig. 2g). We further compared our image-based myeloma quantification with the sample-matched cytological and histological plasma cell counts performed in clinical routine. PCY-based myeloma cell abundance measurements were in good concordance with clinical evaluation of plasma cell infiltration into the BM by cytology (*R*_Sp_ = 0.7; *P* < 2 × 10^−13^; Fig. 2f), which is on par with the similarity between clinical pathology and clinical cytology (*R*_Sp_ = 0.74; *P* < 6 × 10^−10^; Extended Data Fig. 2h). As expected, newly diagnosed patients showed a high abundance of myeloma cells by PCY that were strongly reduced after treatment and re-emerged at relapse (Fig. 2g). Furthermore, sequential samples from patients achieving a partial response (PR) or better to their concurrent clinical treatments showed a decrease in myeloma cell abundance by PCY (Fig. 2h). Thus, the phenotypic signature of myeloma cells and the associated deep-learning strategy for their detection from high-throughput microscopy images forms a clinically concordant basis for quantitative single-cell resolved ex vivo drug screening.

### Distinct bone marrow communities reflect clinical stage

**Fig. 3:**
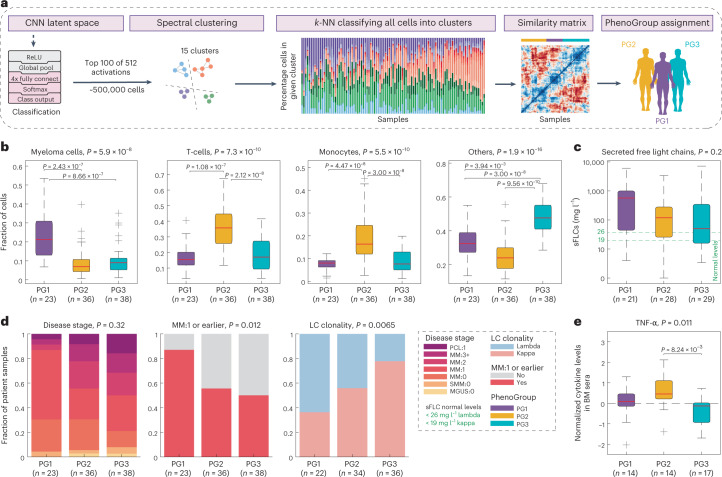
Bone-marrow composition reflects clinical stage, disease clonality and inflammation. **a**, Scheme for determining the PGs. Top 100 activations from the ResNet CNN latent space for the 489,753 cells depicted in Fig. 2a are analyzed by spectral clustering (*k* = 15; Extended Data Fig. 3a) and the remainder of cells were *k*-NN-classified into respective spectral clusters. Sample composition (based on dimethylsulfoxide (DMSO) control cells) is calculated (Extended Data Fig. 3b) and a sample similarity matrix is calculated by correlating spectral cluster abundance per sample. The sample similarity matrix reveals three predominant PhenoGroups revealed by dendrogram cutting (PG1 = purple, PG2 = yellow, PG3 = blue). **b**, Box-plots indicating the fraction of cells per sample, split by cell class and sample PG (for a total of *n* = 97 patient samples). Box-plots as in Fig. 2c. *P* values were calculated using ANOVA. Pairwise *P* values derived from multiple pairwise comparison of the group means using Tukey’s honestly significant difference criterion. **c**, Box-plots showing the clinically measured sFLCs matched to each sample, shown per PG (*n* = 78 patient samples). Green dashed lines represent normal levels (<26 mg l^−1^ for Lambda and <19 mg l^−1^ for Kappa). The *P* value depicted was calculated as in **b**, box-plots as in Fig. 2c. **d**, Distribution of selected clinical parameters across PhenoGroups: fraction of patient samples with selected features are shown as stacked bar graphs. *P* values are calculated using a chi-squared test of independence. For disease stage abbreviations, see legend in Fig. 1b (*n* = 97 patient samples). **e**, Box-plot of *z* score normalized cytokine levels of TNF-α in patient BM sera per PG. The *P* values depicted were calculated as in **b**. Box-plots as in Fig. 2c (*n* = 45 patient samples). Source data

**Extended Data Fig. 3:**
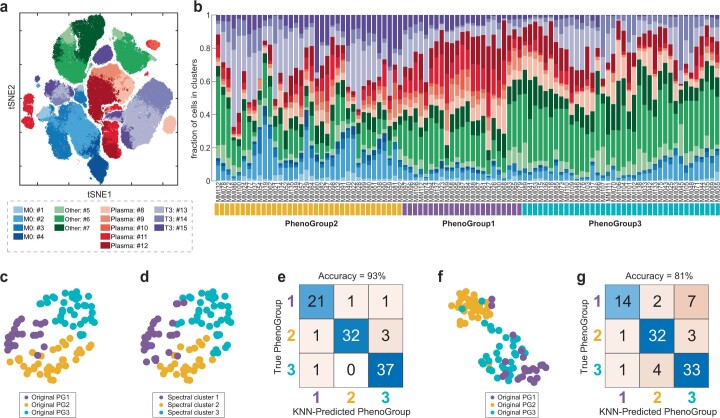
Discovery and robustness of PhenoGroups. **a, left** t-SNE embedding of 489,753 cells (as in Fig. 2a), colored by their respective spectral cluster IDs as detected in top-100 activations of the last fully connected layer of the CNN architecture (‘activation space’). **b**, Stacked barplot showing fraction of cells from DMSO conditions in each spectral cluster per patient sample. Lower colors indicate the corresponding PhenoGroups, as identified by hierarchical tree-cutting. **c**, t-SNE embedding of the 15-class abundance correlation matrix per patient sample. Each dot represents a patient sample colored by their original PG as in **b**. **d**, t-SNE embedding as in **c**, but colored by their Spectral cluster number after spectral (graph) clustering the 15-class abundance correlation matrix with k = 3. **e**, Confusion matrix depicting the accuracy of the KNN-prediced PG of each sample in the 15-class correlation matrix space. The KNN prediction accuracy is 93%. **f**, As in **c**, but calculating the embedding of the 4-class (plasma cells, monocytes, T cells, others) abundance correlation matrix. **g**, As in **e**, but calculating the KNN-predicted PGs in the 4-class abundance correlation matrix space. The KNN prediction accuracy is 81%. **c-g**, N = 97 patient samples. Source data

Imaging millions of BMNCs of each patient sample provides the opportunity to analyze the cellular BM composition across the patient cohort. To capture the cellular heterogeneity identified by the latent space of the four-class CNN, we used graph clustering on the latent-space features to identify phenotypic cell subpopulations (Fig. 3a and Extended Data Fig. 3a). Comparing the cellular composition of the BM samples across these subclasses (Extended Data Fig. 3b) revealed three, surprisingly simple, predominant sample composition modes, which we refer to as PhenoGroups (PGs). Myeloma cells were highest abundant in samples of PG1, T cells and monocytes in PG2 and ‘other’ cells in PG3 (Fig. 3b). We confirmed that the identification of these three PGs was robust to different clustering methods (Extended Data Fig. 3c–e) and supported by the four-class CNN abundances (Extended Data Fig. 3f,g).

**Extended Data Fig. 4:**
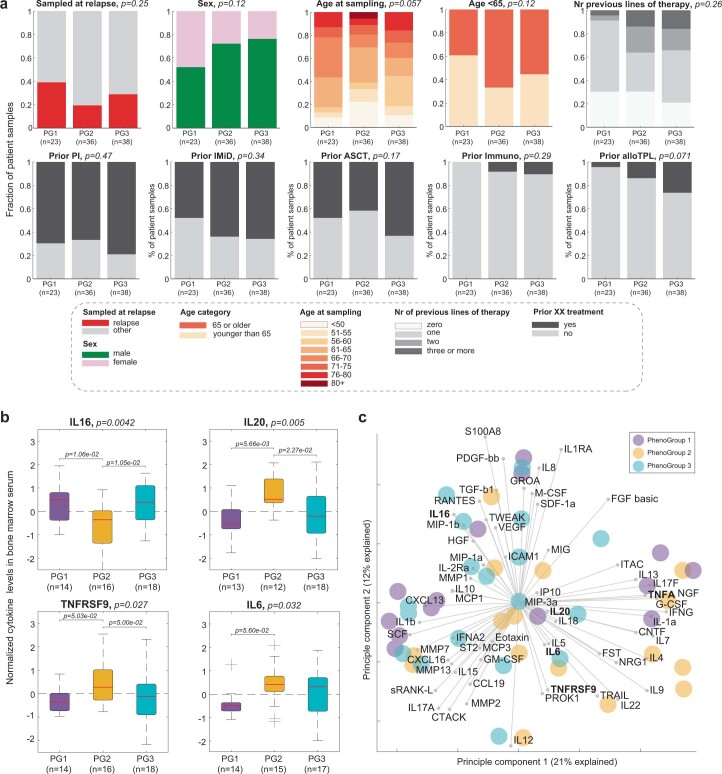
PhenoGroup-associated clinical and cytokine parameters. **a**, Fraction of patient samples per PhenoGroup for not significantly associated clinical features. P-values were calculated using a Chi-square test of independence. Color-coded grouping variables are explained in the legend. **b**, Normalized cytokine levels of additional selected pro-, and anti-inflammatory cytokines measured in patient bone marrow sera. Boxplots (as in Fig. 2c) with normalized cytokine abundance across samples per PhenoGroup are shown. P-values depicted in the titles are calculated using one-way ANOVA, the asterisks indicate p-values from multiple pairwise comparisons of the group means using Tukey’s honestly significant difference criterion. **c**, A principle component analysis (PCA) biplot showing scores of samples (colored by their representative PhenoGroup) with projected loadings (cytokines). **a-b**, N listed in each panel indicates the number of patient samples. Source data

Given the dynamic coevolution of cancer and immune cells in the BM niche in myeloma^12^, we analyzed the clinical associations with the identified PGs (Fig. 3c,dk,l and Extended Data Fig. 4a). Serum-free light chains (sFLCs) in blood, an indicator of active disease^56^, were highest in patients with samples from PG1, consistent with their high myeloma cell abundance (Fig. 3c). PG1 was significantly enriched in samples from patients who have received up to one previous line of therapy. In contrast, more pretreated patients dominated in PG2 and PG3 (Fig. 3d, left and middle). Light-chain clonality strongly associated with the PGs, with Lambda clones representing over 60% of samples in PG1, whereas over 70% of samples in PG3 were Kappa clones (Fig. 3d, right). Other clinical parameters, including patient age and sex, genetic aberrations and the type of previous therapy line, did not associate with the PGs (Extended Data Fig. 4a). Thus, the three distinct cellular communities detected across the cohort reflected different disease-associated processes, active disease in PG1 and high immune cell infiltration in PG2, with associations to both the patient’s treatment stage and clonality of the disease.

Complexity of the BM environment in MM results from dynamic changes in cell composition^12,57^ and the interaction of myeloma cells with the surrounding stromal cells^11^. We therefore further analyzed the PG signatures by systematic cytokine profiling in BM sera of 48 selected samples (Fig. 3e, Extended Data Fig. 4b,c and Supplementary Table 2). Principal-component analysis and analysis of variance (ANOVA) on normalized cytokine abundances revealed that the immune-infiltrated samples from PG2 were particularly high in proinflammatory cytokines, including TNF-α and IL-6 (refs. ^13,14^), whereas PG1 was notably low in IL-6 (Fig. 3e and Extended Data Fig. 4b,c). Thus, both the image-based cell composition and cytokine profiling are consistent with the presence of three predominant BM states among our patient cohort that dynamically change during the disease course.

### The myeloma cell proteotype

**Fig. 4:**
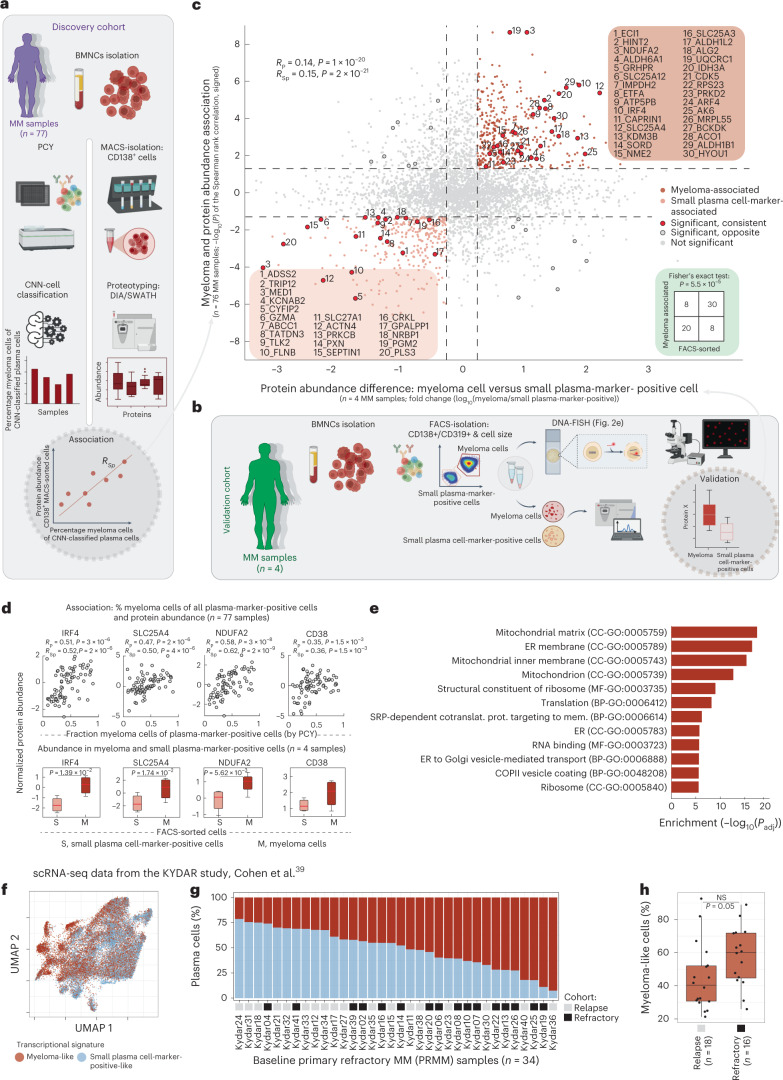
The molecular proteotype of myeloma cells. **a**, Work scheme for the integration of proteotype and PCY data on the discovery cohort (*n* = 77 patient samples). **b**, Work scheme for validation cohort (*n* = 4 patient samples; also analyzed in Fig. 2e). **c**, Scatter-plot. The *y* axis represents myeloma and protein abundance associations calculated as in **a** as signed *P* values of Spearman’s rank correlations. The *x* axis represents the difference in protein abundance between FACS-sorted myeloma and small plasma cell-marker-positive cells from four myeloma samples (scheme **b**). Top-right quadrant shows proteins positively associated with myeloma cells across the discovery cohort (*n* = 77) and upregulated in myeloma cells in the validation cohort (*n* = 4). Lower-left quadrant contains negatively associated with and downregulated proteins in myeloma cells. Gray dashed line represent the cutoffs for *y* axis (*P* < 0.05) and *x* axis (absolute fold change > 0.3). Fisher’s exact test insert indicates significance in overlap of proteins, significant in both discovery and validation cohorts (*P* < 5.47 × 10^−5^). Selected proteins are numbered and protein identifiers reported in the respective quadrants. Proteins formatted in bold letters are shown in **d** (see also Supplementary Table 4). Spearman’s rank and Pearson’s correlations and *P* values are indicated (top left). **d**, Example data for myeloma-associated proteins IRF4, SLC25A4, NDUFA2 and CD38. Top scatter-plots indicate protein abundance (*y* axis) against percentage myeloma by PCY (*x* axis) (*n* = 77 patient samples). Bottom box-plots (as in Fig. 2c) show protein abundance (*y* axis) per FACS-sorted myeloma (M) and small plasma cell-marker-positive cells (S) cells of the validation cohort (*n* = 4 patient samples). *P* values from a paired two-tailed Student’s *t*-test. Pearson’s and Spearman’s rank correlation and associated *P* values are provided under the title of each upper plot. *P* values not corrected for multiple testing. **e**, Gene Ontology enrichment analysis of the myeloma protein signature. *P* value by hypergeometric test, false discovery rate (FDR)-adjusted for multiple comparisons. SRP, signal recognition particle. **f**, Uniform Manifold Approximation and Projection representation of scRNA expression levels^39^ colored by their myeloma-like and small plasma cell-marker-like transcriptional signature (*n* = 31,305 cells from 34 patients). **g**, Bar-plots of the percentage plasma cells colored as in **f** (*n* = 34 patients). **h**, Box-plots (as in Fig. 2c) show percentage myeloma-like cells per relapse (*n* = 18 patients) and refractory (*n* = 16 patients) disease stages. Indicated *P* value (not significant) is from an unpaired two-tailed Student’s *t*-test. Source data

**Extended Data Fig. 5:**
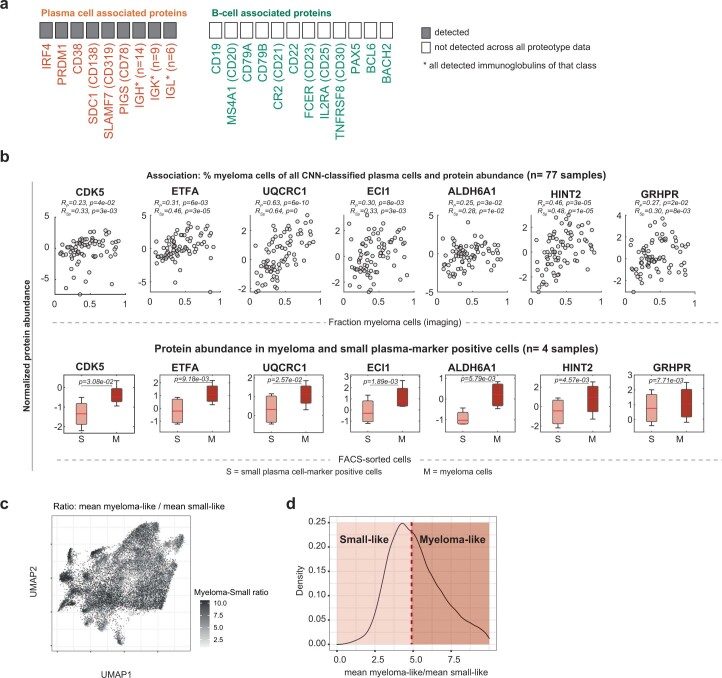
Molecular investigation of the myeloma signature. **a**, Detected plasma cell associated proteins, and not-detected B-cell associated proteins, as measured by DIA-SWATH proteomics in the MACS-sorted CD138^+^ plasma cell samples across the entire cohort (N = 77 patient samples). **b**, Scatterplots of additional example proteins showing high association with the percentage of myeloma cell morphology of CNN-detected plasma cells in the discovery cohort (n = 77 samples) (upper panel), which are confirmed in FACS-sorted cells of the validation cohort (lower panel; boxplots as in Fig. 2c). Dots represent measurements from individual patient samples. Spearman’s rank and Pearson’s correlations and p-values are indicated. Below, p-values indicated from a paired two-tailed t-test (n = 4 patient samples), not adjusted for multiple testing. **c**, UMAP representation of the KYDAR study’s single-cell transcriptional profiles of sorted plasma cells^39^. Dots are colored by the expression ratio of myeloma-associated over small plasma cell-marker-positive-associated genes as identified in the upper right and lower left quadrants of Fig. 4c. **d**, Density histogram of transcriptional signature ratios across cells, with a threshold for transcriptional signature at the median myeloma-to-small ratio of 5. Source data

With the aim to identify the molecular drivers of drug response in MM, we characterized the proteotype of myeloma cells. Because of the heterogeneity among CD138^+^ enriched cells in MM (Fig. 2a–e), we followed two complementary strategies: (1) Where the cell numbers allowed it, across the (discovery) cohort we performed proteotyping analysis of plasma cells enriched by their CD138 surface expression using MACS for 77 patient samples (Supplementary Table 3). For the 5,663 detected proteins, we next calculated their abundance association with the sample-matched fraction of myeloma cells relative to all plasma cell-marker-positive cells as measured by PCY (Fig. 4a). Thus, proteins that score positively by this approach should be more abundant in myeloma cells, whereas proteins that score negatively should be less abundant in myeloma cells. (2) Complementary to this discovery approach, we performed validation measurements in the genetically characterized flow-sorted CD138^+^/CD319^+^ big (myeloma; mostly hyperdiploid) and small (mostly diploid) cells from four patients (Fig. 4b, Extended Data Fig. 2e and Fig. 2e). Of note, both discovery and validation proteotyping detected numerous plasma cell-associated proteins, even for the mostly diploid small plasma cell-marker-positive cells, whereas common B-cell markers were not detected (Extended Data Fig. 5a). When comparing the results of these two approaches, we observed significant consistency between the myeloma-associated proteins of the discovery cohort and the differentially expressed proteins of the validation measurements (*P* < 1 × 10^−20^ by Spearman’s rank correlation; *P* < 5.5 × 10^−5^ by Fisher’s exact test; Fig. 4c). The resulting myeloma proteotype signature (top right quadrant in Fig. 4c, consisting of 411 proteins; Supplementary Table 4) aligned well with known features of MM; notable was the high expression of IRF4 in myeloma cells, a master regulator of plasma cell differentiation and a key driver of MM^8^ (Fig. 4c,d). Gene Ontology enrichment analysis and additional investigation of the myeloma proteotype signature highlighted high abundance of the antibody secretory pathway, as well as of mitochondrial, ribosomal, proteasomal and translational machinery^37,39^ (Fig. 4e). The identified myeloma proteotype further suggested promising and recently investigated myeloma drug targets, including CDK5 (ref. ^58^) (Extended Data Fig. 5b) and endoplasmic reticulum (ER)-stress associated HSPA5 and HSP90B1 (ref. ^59^) (Supplementary Table 4).

We confirmed the presence of the myeloma proteotype signature in single-cell RNA sequencing (scRNA-seq) of MM patient samples, re-analyzing publicly available data of the KYDAR study^39^. We calculated the ratio of the mean expression of detected genes from either the myeloma signature or from the healthy/small plasma cell-marker-positive cell signature genes (bottom left quadrant in Fig. 4c). We classified more myeloma-like cells based on their highly increased expression of the myeloma signature (ratio > 5; Fig. 4f and Extended Data Fig. 5c,d). Notably, across the 34 patients with relapsed or refractory MM analyzed at baseline in the KYDAR study, the myeloma-like cells tended to be more abundant in refractory patients who failed to respond to a bortezomib-containing first-line treatment (Fig. 4g,h)^39^. Thus, the myeloma proteotype signature was associated with aggressive disease in scRNA-seq analysis of patients with MM.

### The MM drug response landscape reveals patient variability

**Fig. 5:**
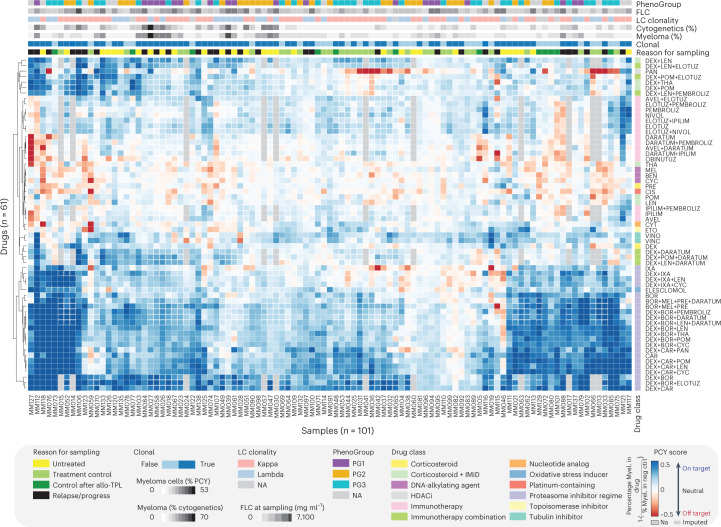
The single-cell drug response landscape of MM. Myeloma cell drug responses (PCY scores) bi-clustered across the cohort of 101 patient samples (columns) and 61 unique drugs and drug combinations (rows). Additional sample and drug annotations are provided at the top and right of the drug response matrix (see legend). For visualization purposes, 21% (1,292 of 6,161) of shown drug responses have been imputed by LASSO regression on either measured drug responses or matching myeloma proteotype data. Imputed drug responses are indicated by a dashed outline. LC, light chain, AVEL, avelumab; BEN, bendamustine; BOR, bortezomib; CAR, carfilzomib; CIS, cisplatin; CYC, cyclophosphamide; CYT, cytarabine; DARA, daratumumab; DEX, dexamethasone; ELOTUZ, elotuzumab; ETO, etoposide; IPILIM, ipilimumab; IXA, ixazomib; LEN, lenalidomide; MEL, melphalan; NIVOL, nivolumab; OBINUTUZ, obinutuzumab; PAN, panobinostat; PEMBROLIZ, pembrolizumab; POM, pomalidomide; PRE, prednisone; THA, thalidomide; VINC, vincristine; VINO, vinorelbin. Source data

**Extended Data Fig. 6:**
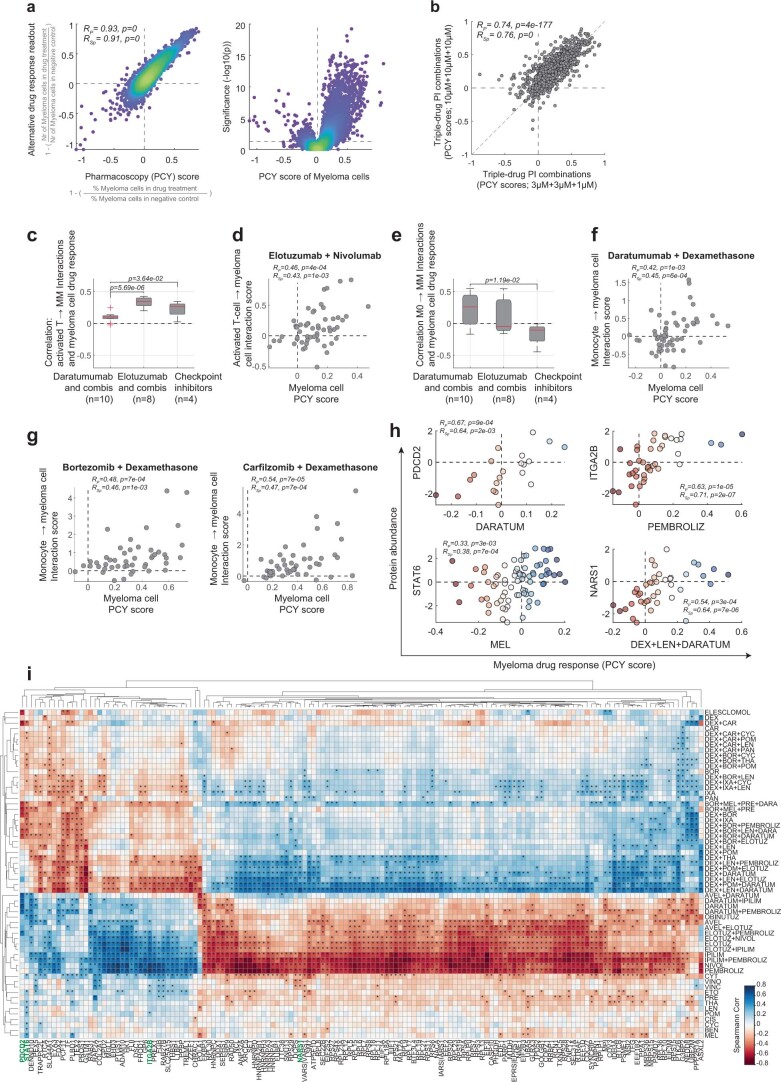
Molecular network underlying the single-cell drug response landscape of myeloma. **a, left**, Scatter plot showing the similarity between PCY scores of myeloma cells as used throughout this study (x-axis; calculated as (1 - (% of myeloma cells in drug treatment divided by % myeloma cells in matched control conditions))) and drug response scores based on the number of myeloma cells relative to their number in matched control conditions (y-axis; thus independent of the drug response of healthy monocytes, T cells and other cells in each sample). The scatter plot shows all measured patient samples and drug conditions. Spearman’s rank and Pearson’s correlations and p-values are indicated. (N = 6161, that is 61 drug perturbations times 101 patient samples). **a, right**, Volcano plot of all measured myeloma PCY scores and their corresponding significance as measured by Student’s t-test against the relevant control wells. **b**, Scatter plot showing the consistency in *ex vivo* drug responses to the lowest (x-axis) and highest (y-axis) concentrations of the triple-drug proteasome inhibitor regimes tested across the cohort (N = 6161, that is 61 drug perturbations times 101 patient samples). Spearman’s rank and Pearson’s correlations and p-values are indicated. **c**, Boxplots showing the distribution of Spearman’s rank correlations (R_Sp_) between the activated T cell to myeloma cell Interaction score and the myeloma cell drug responses across all measured samples (N = 56). R_Sp_ values are aggregated across Daratumumab containing treatments (n = 10 treatments), Elotuzumab containing treatments (n = 8 treatments), and the four individually tested immune checkpoint inhibitors (n = 4 treatments). Boxplots as in Fig. 2c. **d**, Example scatter plot of the activated T cell to myeloma cell interaction score (y-axis) and the myeloma cell drug responses (x-axis) across all measured samples (n = 55) for the combination of Elotuzumab and Nivolumab. Spearman’s rank and Pearson’s correlations with p-values are indicated. **e**, as in **c**, but for monocyte to myeloma cell interactions. **f**, as in **d**, but for monocyte to myeloma cell interactions for the combination of Daratumumab and Dexamethasone (n = 56 patient samples). Spearman’s rank and Pearson’s correlations with p-values are indicated. **g**, as in **f**, but for either Bortezomib and Dexamethasone in combination (left) (n = 47 patient samples), or Carfilzomib and Dexamethasone in combination (right) (n = 48 patient samples). Spearman’s rank and Pearson’s correlations with p-values are indicated. **h**, Examples of four associations between myeloma drug response (PCY score; x-axis) and myeloma protein abundance as measured by DIA-SWATH proteomics (y-axis). Spearman’s rank and Pearson’s correlations with p-values are indicated. DARATUM to PDCD2 association: n = 21 patient samples; PEMBROLIZ to ITGA2B association: n = 41 patient samples; MEL to STAT6 association: n = 76 patient samples; DEX + LEN + DARATUM to NARS1 association: n = 41 patient samples. **i**, Clustergram of protein-drug associations (Spearman’s rank coefficients across the cohort) for the 150 proteins with most variable associations across the drug library. Stars indicate R_Sp_ with p < 0.05. Source data

To get an overview of the drug response differences between patients with MM, we next measured the ex vivo myeloma cell responses of 101 BM samples by PCY to a panel of 61 drugs and drug combinations (Supplementary Tables 5 and 6, Fig. 5 and Extended Data Fig. 6a,b). We quantified the level of on- and off-target drug efficacy after 24 h of incubation by contrasting the ex vivo drug response of myeloma cells with that of healthy cells of the MM BM samples present in the assay. Positive PCY scores indicate on-target efficacy, which is more extensive death of myeloma cells compared to the patient’s own healthy cells, whereas negative PCY scores indicate off-target toxicity, which is more extensive death of healthy cells compared to the myeloma cells. The PCY scores showed excellent technical reproducibility and strongly correlated with myeloma cell numbers relative to control (Extended Data Fig. 6a). The latter indicates that the myeloma PCY scores were predominantly determined by drug-induced changes in the viability of the myeloma cells, not healthy cells, simplifying the interpretation of the results. Furthermore, we observed that the PCY-based drug responses were reproducible across different concentrations (Extended Data Fig. 6b and Supplementary Table 5). Clustering the drug responses across the cohort further revealed strong similarity in the responses to drugs and drug combinations of the same class, resulting in the grouping of drug class annotations (Fig. 5). The emerging drug response landscape revealed extensive variability between patients (Fig. 5). Across the clinically oriented drug panel, PI-containing treatments were active in the largest fraction of patient samples, as expected based on their repeated use in the treatment of MM; however, notable differences were observed, with subsets of patient samples responding well to all three tested PIs, whereas others responded ex vivo to none (with PCY scores close to 0). Top drug combinations across the cohort included the combination of the corticosteroid dexamethasone with either bortezomib or carfilzomib, which form the backbone of non-immunotherapy-based clinical treatments for MM.

Furthermore, we observed considerable sensitivity to approved and experimental immunotherapies for MM, albeit with reduced response depths compared to tested PI-containing treatments, likely explained by their different dynamics and mode of action. Immunotherapies with ex vivo on-target responses (PCY > 0) included daratumumab (targeting CD38) and elotuzumab (targeting CD319/SLAMF7), both clinically approved for MM^18–21^. Subsets of samples responded well to experimental immunotherapy combinations, including combinations containing checkpoint inhibitors nivolumab and pembrolizumab (both targeting PD-1), avelumab (targeting PDL-1) and ipilimumab (targeting CTLA-4). As expected, obinutuzumab, which targets B-cell marker CD20, was among the least active ex vivo tested drugs, consistent with the absence of CD20 and other B-cell marker expression by proteotyping (Extended Data Fig. 5a). Such ex vivo responses to targeted immunotherapies and checkpoint inhibitors imply immune cell activation and engagement between effector cells and myeloma cells. Both can be analyzed by measuring cell morphology and cell–cell contacts in our image data^50–52^. Engagement of activated T cells with myeloma cells particularly associated with ex vivo response to elotuzumab and combinations thereof, as well as with response to the immune checkpoint inhibitors (Extended Data Fig. 6c,d). In contrast, ex vivo response to daratumumab and dara-combinations were associated with engagement between monocytes and myeloma cells (Extended Data Fig. 6e,f), in line with previous reports on the involvement of monocytes in the response to daratumumab^60,61^. Immune cell involvement in drug responses, however, seemed to go beyond responses to immunotherapies. For instance, ex vivo response to the strongest drug combinations, dexamethasone with either bortezomib or carfilzomib, associated with increased interactions between monocytes and myeloma cells (Extended Data Fig. 6g). Thus, our ex vivo drug response measurements incorporated the patient’s own immune cells, informing on differential involvement of effector cells and revealing extensive patient heterogeneity in drug sensitivity and resistance.

### The molecular network underlying the drug response landscape

**Fig. 6:**
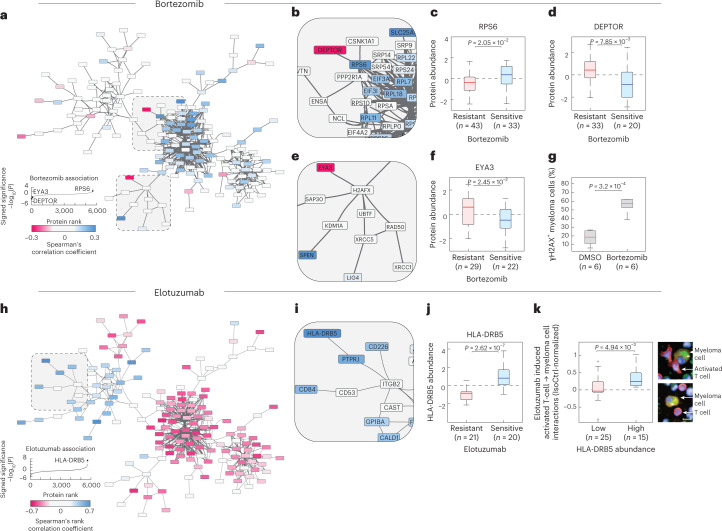
The protein network underlying MM drug sensitivity. **a**, STRING-db interaction network for the proteins whose abundance in myeloma cells most strongly correlates with myeloma drug responses across all proteins and drugs. Unconnected nodes in the network are not shown for simplicity. Node color represents the Spearman’s rank correlation with bortezomib response across the discovery cohort. More details are at https://myelomics.com. **b**, Zoom into the network region around RPS6 and DEPTOR. Legend shows signed −log_10_(*P*) per protein, sorted by their Spearman’s rank correlation coefficients. No adjustments for multiple comparisons were made. **c**, Box-plots (as in Fig. 2c) showing RPS6 abundance as a function of bortezomib sensitivity (*n* = 76 patient samples). **d**, Box-plots (as in Fig. 2c) showing DEPTOR abundance as a function of bortezomib sensitivity (*n* = 53 patient samples). **e**, Zoom into the network region around EYA3. **f**, Box-plots (as in Fig. 2c) showing EYA3 abundance as a function of bortezomib sensitivity (*n* = 51 patient samples). **g**, Box-plots showing the percentage of γH2AX-positive myeloma cells after 24 h of DMSO or bortezomib treatment across six newly diagnosed myeloma samples (*n* = 6 patient samples). Box-plots as in Fig. 2c. *P* values shown are from a two-tailed paired Student’s *t*-test. **h**, STRING-db interaction network colored by their elotuzumab drug response associations, as in **a**. **i**, Zoom into the network region around HLA-DRB5. **j**, Box-plots (as in Fig. 2c) showing HLA-DRB5 abundance as a function of elotuzumab sensitivity (*n* = 41 patient samples). **k**, Box-plots (as in Fig. 2c) showing the elotuzumab-induced activated T cell interactions with myeloma cells as a function of HLA-DRB5 abundance (*n* = 40 patient samples). Example images on the right of the plot show an activated T cell with close contact to a myeloma cell (top) and a conventional T cell without cell–cell contact to a myeloma cell (bottom). Scale bar, 10 μm. All *P* values depicted on box-plots (**c**,**d**,**f**,**g**,**j**,**k**) are from unpaired two-tailed Student’s *t*-tests. The tests were not adjusted for multiple comparisons. All box-plots (**c**,**d**,**f**,**g**,**j**,**k**) as in Fig. 2c. Source data

To identify the molecular states associated with these differential drug responses, we correlated the drug responses with myeloma protein abundances across the discovery cohort (Extended Data Fig. 6h,i and Supplementary Table 7). We further identified key predictor proteins of drug response using regularized (elastic net) regression (Supplementary Table 7). Clustering the top variable drug–protein correlations revealed a notable bimodal pattern, with PIs, dexamethasone, elesclomol and panobinostat-containing treatments globally following one protein association pattern, whereas immunotherapies, IMiDs (lenalidomide and pomalidomide) and chemotherapies followed the opposite protein association pattern (Extended Data Fig. 6i). As protein abundance of co-regulated proteins is often correlated, we analyzed these protein–drug response associations in the context of their known regulatory network (as defined by the STRING database^62^) (Fig. 6a). Visualizing the core network of strongest drug-associated proteins across all of the 61 drugs and drug combinations revealed well-defined functionally related protein subnetworks, centered around a ribosomal and translational core network (including, for example, RPS6, DEPTOR, EEF1A1 and EIF3D) connected to messenger RNA splicing (including SRSF2, SRSF6 and SRSF9), tRNA synthesis (EPRS, NARS and SARS), DNA repair (including H2AX, EYA3, XRCC5 and RAD50), protein ubiquitination and degradation (including PSMD7, PDMB10, NEDD8 and UBE2M) and a more diverse signaling and cell adhesion subnetwork (including STATs, integrins, ER-resident chaperones and HLA-DRB5, among others) (Fig. 6a). Visualizing the associations with any one drug on this network highlighted striking contextual relationships. For example, a top negative association to bortezomib was DEPTOR^63^, which connected to the strongly positively associated RPS6 (Fig. 6b). These opposite yet connected associations are in line with RPS6 being regulated downstream of mTOR and S6K signaling, whereas DEPTOR is a negative regulator of mTOR. Combined, these associations suggest a bortezomib-sensitive MM state that is DEPTOR-low and RPS6-high, further characterized by high abundance of ribosomal, translational and mRNA splicing machinery (Fig. 6b–d).

**Extended Data Fig. 7:**
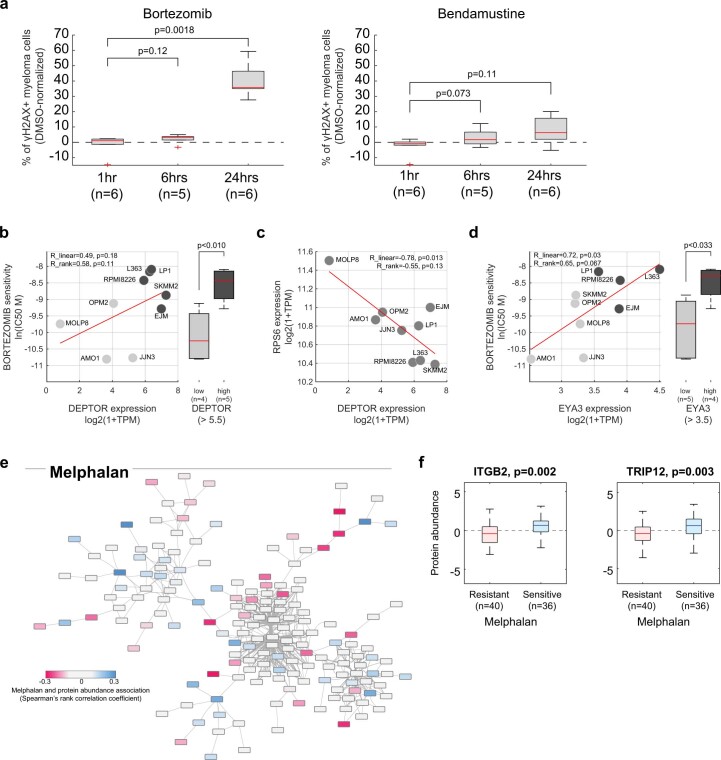
Molecular determinants of myeloma drug sensitivity. **a**, Boxplots showing percentage of ɣH2AX-positive myeloma cells, normalized to levels in DMSO (by subtraction), after different incubation times with 1 μM Bortezomib (**a, left**), or 1 μM Bendamustine (**a, right**) (n in each panel indicates the number of patient samples). Boxplots as in Fig. 2c. P-values of paired two-sided t-tests between condition and DMSO are shown. **b**, Scatter plot of Bortezomib sensitivity (ln(IC50)) versus *Deptor* transcript abundance (log_2_(1+TPM)) for MM cell lines analyzed in^66^, data accessed through DepMap portal (https://depmap.org) (**b, left**). R_linear is Pearson’s linear, and R_rank is Spearman’s rank correlation coefficient, with indicated p-values. MM cell line names are labeled (n = 9 cell lines). **b, right**, Boxplots comparing Bortezomib sensitivity for *Deptor* low and high MM cell lines. P-value by unpaired two-tailed Student’s t-test. Boxplots as in Fig. 2c. (n = 4 DEPTOR-low cell lines, n = 5 DEPTOR-high cell lines). **c**, Scatterplot comparing *Deptor* and *Rps6* expression levels in MM cell lines of **b**. Spearman’s rank and Pearson’s correlations with p-values are indicated. **d**, As in **b**, but comparing Bortezomib sensitivity to *Eya3* transcript abundance. Spearman’s rank and Pearson’s correlations with p-values are indicated. The p-value on boxplot is by unpaired two-tailed Student’s t-test. **e**, STRING-db network of top drug-associated proteins as in Fig. 4b colored by Melphalan drug response association. Color represents the sign and strength of association (color code as legend in Fig. 4b). Please see https://myelomics.com for more network details. **f**, Protein abundance of ITGB2 (**left**) and TRIP12 (**right**), positively associated with Melphalan sensitivity, are shown. The depicted p-value is by unpaired two-sided Student’s t-test (n = 76 patient samples). Boxplots as in Fig. 2c. Source data

The second strongest negative bortezomib association captured in the network analysis was EYA3, part of the DNA repair subnetwork (Fig. 6e,f). EYA3 is a phosphatase of tyrosine-142 phosphorylated histone H2AX^64^. H2AX phosphorylation, including phosphorylated serine-139 (γ-H2AX), is a hallmark of genotoxic stress^65^. As such, EYA proteins are key regulators of the survival decision in response to genotoxic stress and DNA damage^64^, although they have to date not been implicated in the response to bortezomib in MM. Bortezomib treatment is known to reduce DNA repair capabilities in MM^28^ and bortezomib-resistant MM samples in our cohort showed increased EYA3 expression levels, suggesting increased EYA3 expression might help MM cells survive bortezomib-induced genotoxic stress. In line with this interpretation, bortezomib strongly induced γ-H2AX accumulation after 24 h of treatment in myeloma cells across six validation samples of newly diagnosed patients (Fig. 6g), notably higher than the γ-H2AX levels induced in the same samples by the DNA-damaging agent bendamustine (Extended Data Fig. 7a). Thus, our data hint toward a role for EYA3 in regulating the survival decision upon bortezomib-induced genotoxic stress in primary myeloma cells.

We tested whether these DEPTOR/RPS6 and EYA3 associations with bortezomib sensitivity observed in primary MM samples were recapitulated in publicly available drug results and transcriptomics of MM-derived cell lines^66^. Indeed, *DEPTOR-*high-expressing cell lines showed increased resistance to bortezomib (Extended Data Fig. 7b). And *DEPTOR* and *RPS6* transcript abundance were significantly anticorrelated (Extended Data Fig. 7c). Furthermore, *EYA3* transcript abundance was associated with increased bortezomib resistance (Extended Data Fig. 7d). Thus, our primary MM sample proteomics and image-based drug screening results could be recapitulated in transcriptomics and bulk-drug screening results on MM-derived cell lines, further supporting a role for both DEPTOR and EYA3 as regulators of bortezomib sensitivity in MM.

We next investigated elotuzumab, a clinically approved SLAMF7/CD319-targeting immunotherapy, as an example of the opposite drug–protein association cluster (Fig. 6h). Elotuzumab was ex vivo more effective against myeloma cells with relatively reduced ribosomal, mRNA splicing and ER chaperone protein abundance (Fig. 6h). The top-ranked positive association was with the major histocompatibility complex class II (MHC-II) subunit DRβ5 (HLA-DRB5) (Fig. 6i,j). While mature plasma cells typically do not express MHC-II molecules, myeloma cells can re-express MHC-II molecules in response to extrinsic cues such as interferon-γ^67^. MHC-II molecules mediate antigen presentation to CD4^+^ T cells, leading to cell–cell contacts that can be quantified by PCY^50–52^. Across the cohort, we observed increased interactions between myeloma cells and activated T cells upon elotuzumab treatment for HLA-DRB5-high MM samples compared to HLA-DRB5-low samples (Fig. 6k), suggesting that MHC-II-mediated antigen presentation associates with increased sensitivity to elotuzumab.

The pan-drug–protein association analysis suggested a molecular rationale for existing clinical treatment regimes. For example, chemotherapy, such as melphalan and immunotherapies often follow PI-containing treatments in the clinic and seemed to target complementary proteotypic states of myeloma cells in our analysis (Extended Data Fig. 6i). Melphalan’s protein associations were highly distinct from those of bortezomib (Extended Data Fig. 7e), with increased efficacy in myeloma cells with high expression levels of integrin B2 (ITGB2) and E3 ubiquitin-protein ligase TRIP12, among others (Extended Data Fig. 7f). Results from other protein–drug networks can be visualized and accessed at https://myelomics.com.

### Treatment strategies for clinically defined subcohorts

**Fig. 7:**
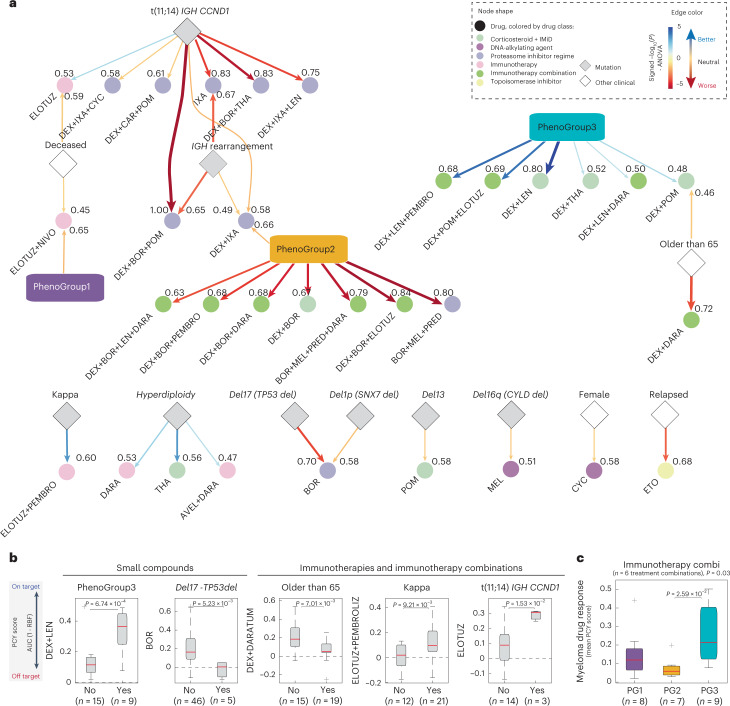
Therapeutic strategies for clinically defined myeloma subcohorts. **a**, Network representing clinical and morphological features associated with myeloma drug sensitivity both by ANOVA analysis as well as by a two-tailed Student’s *t*-test across *n* = 67 patient samples. Drugs are represented as circles, colored by their respective drug class. Numbers next to the arrows indicate fraction of times the association is significant in cross-validation. Mutations and other clinical parameters are represented in rhombus shapes and PGs in rectangular shapes in their respective colors. Edge color shows better (blue) or worse (red) sensitivity toward a drug or a drug combination for a group of patients. Drugs and drug combinations are indicated by shortened drug names concatenated by a + symbol (Supplementary Table 5). **b**, Box-plots (as in Fig. 2c) showing example differences in PCY scores for distinct patient subsets and drugs, associated with **a**. Indicated *P* values are from an unpaired two-tailed Student’s *t*-test; *n* values indicate number of samples with a selected feature and a measured PCY response. **c**, Box-plots (as in Fig. 2c) showing the distribution of mean PCY responses per sample across six drugs in the immunotherapy combinations class, grouped by PG (*n* = 24 patient samples). One-way ANOVA *P* value is reported, the asterisk indicates a *P* value from multiple pairwise comparison of the group means using Tukey’s honestly significant difference criterion. Source data

**Extended Data Fig. 8:**
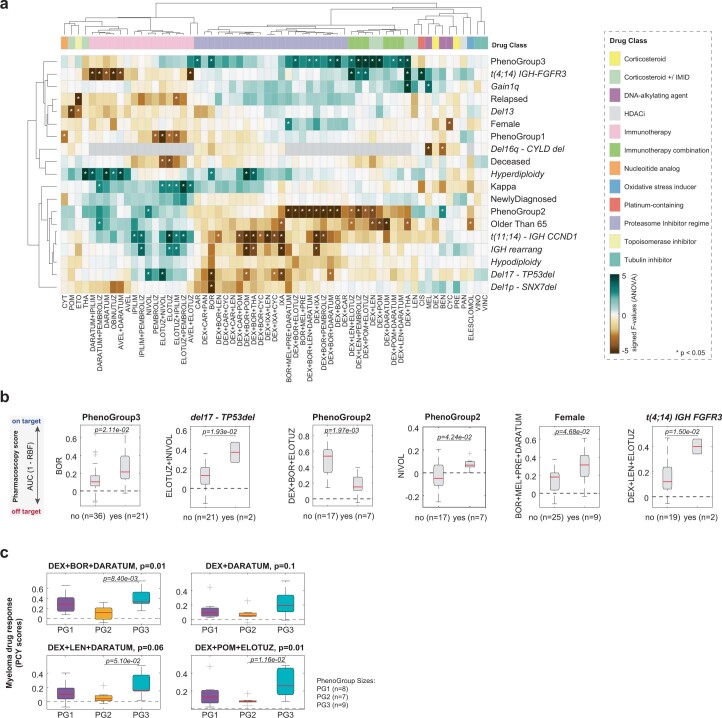
Clinical parameters associated with drug sensitivity. **a**, Heatmap showing signed F-values from one-way ANOVAs on drug responses for different clinical and morphological features across the cohort, calculated on clonal patient samples (n = 67). Positive values indicate a feature contributing to a better PCY outcome (sensitivity), whereas negative values indicate association with worse PCY score (resistance). * indicate p-values lower than 0.05. Drug classes are annotated by color above the heatmap, see legend. **b**, Boxplots showing differences in Pharmacoscopy scores in patients with a selected clinical or morphological feature. P-values indicate significances out of unpaired two-sided Student’s t-tests. No adjustments for multiple comparisons were made. Boxplots as in Fig. 2c. See figure for numbers of patient samples used. **c**, Selected Pharmacoscopy scores for immunotherapy combinations frequently given in the clinic, shown per PhenoGroup. Numbers of patient samples included in each test are reported. Boxplots as in Fig. 2c. Source data

Our cohort analysis allowed us to explore possible clinical biomarkers to guide treatment decisions in MM. To this end, we integrated genetic, clinical and PG annotations with the ex vivo drug responses for patients with confirmed clonal disease and available clinical data (Fig. 7a and Extended Data Fig. 8a,b). The largest number of associations related to the PGs. PG2, characterized by high monocyte and T cell infiltration and high TNF-α and IL-6 cytokine levels (Fig. 3b,e and Extended Data Fig. 4b,c), responded poorly ex vivo to combinations of bortezomib and corticosteroids (dexamethasone or prednisone) optionally including immunotherapies. PG3, characterized by a high fraction of ‘other’ cells, in contrast, showed significantly higher ex vivo sensitivity to combinations of dexamethasone and IMiDs (lenalidomide, pomalidomide or thalidomide), optionally including immunotherapies, compared to PG1 and PG2 (Fig. 7a,b and Extended Data Fig. 8a,b). The PGs showed significant associations also when grouping the responses to all tested immunotherapy combinations, with samples of PG2 not responding to most immunotherapy combinations ex vivo (PCY scores of around 0), whereas samples of PG3 on average showed the strongest ex vivo responses, suggesting a strong role for the BM microenvironment in influencing response to immunotherapy, revealed by our ex vivo platform (Fig. 7c and Extended Data Fig. 8c).

Beyond the PGs, genetics and patient characteristics also showed exploratory associations to drug response. The common chromosomal translocation t(11;14)(q13;q32), linked to upregulation of cyclin D1 (CCND1)^7^, associated with worse response ex vivo to several less commonly used triple-drug combinations of PIs, steroids and IMiDs, when compared to t(11;14)-negative samples. Significantly higher sensitivity of t(11;14)-positive samples was, however, observed to elotuzumab (Fig. 7a,b). Deletion of the 17p13 chromosomal region [del(17p)] is associated with mutations in *TP53* and poor outcomes in MM^30^. Moreover, bortezomib has been shown to activate and depend on p53 in myeloma cell line models^68,69^. Consistently, with a median PCY score of around 0, the del(17p) mutant samples showed significant ex vivo resistance to bortezomib (Fig. 7a,b), further pointing toward a role for the DNA repair pathway in regulating sensitivity to bortezomib in primary myeloma samples. The combination of elotuzumab with checkpoint inhibitor pembrolizumab resulted in significantly higher on-target PCY scores in MM samples with Kappa-clonal disease; and the clinically used combination of dexamethasone with daratumumab was observed to work best in samples of patients younger than 65 years (Fig. 7a,b). Thus, our results provide a rich resource to explore possible response biomarkers and treatment strategies to both existing and experimental myeloma treatment options.

### Pharmacoscopy stratifies clinical response to immunotherapy

**Fig. 8:**
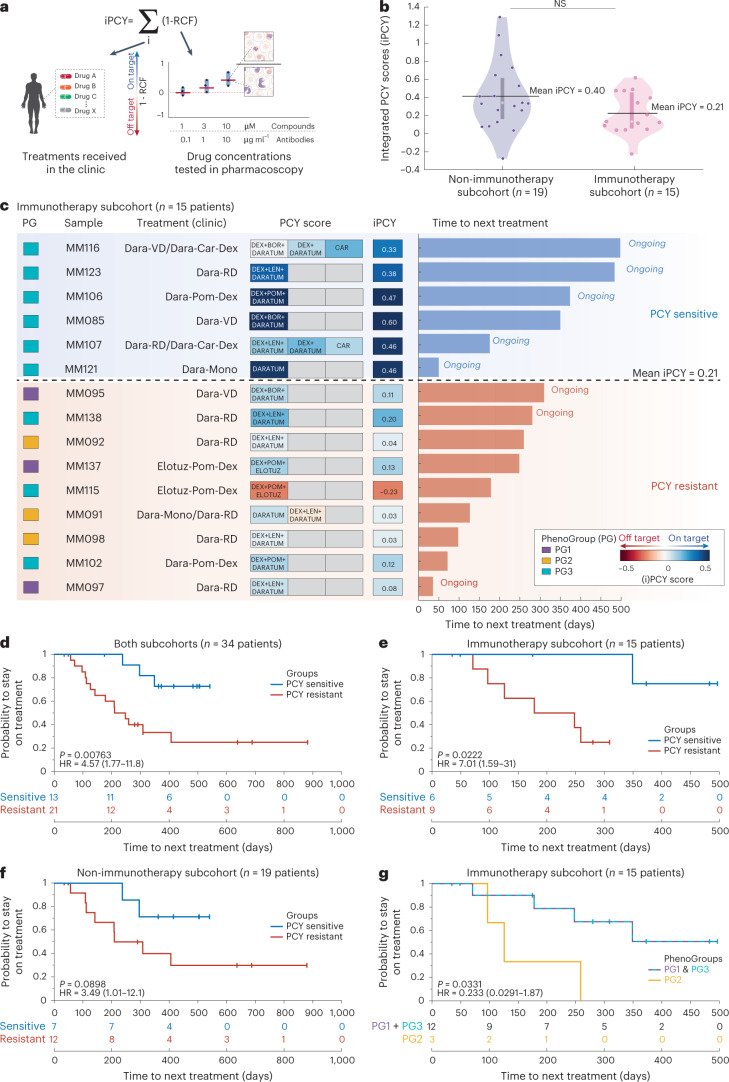
Ex vivo drug sensitivity stratifies clinical responses. **a**, A schematic representation of the iPCY scores: the sum of ex vivo drug responses (1 − relative cell fraction) matching the treatments the patient subsequently received in the clinic. **b**, Violin plots with a depicted median and kernel density estimate of the data of iPCY scores for the non-immunotherapy subcohort (left; *n* = 19 patients) and immunotherapy subcohort (right; *n* = 15 patients). Additionally, mean iPCY of each subcohort is depicted (horizontal black lines) and used as threshold to separate more sensitive (‘PCY-sensitive’) from less sensitive (‘PCY-resistant’) patients. Difference was not significant by unpaired two-tailed Student’s *t*-test. **c**, A graphic representation of the clinical immunotherapy subcohort (*n* = 15 patients). Patients are included based on receiving either daratumumab or elotuzumab-containing treatment following PCY testing and having evaluable response. PG, sample identifier and clinical treatment per patient are reported. Heat map shows the individual PCY scores matching to the treatments given, with their respective iPCYs on the right. Finally, time to next treatment is reported per patient, with blue indicating PCY-sensitive and red PCY-resistant samples as in **b**. **d**, Kaplan–Meier curve for the probability to stay on treatment for both subcohorts combined (*n* = 34 patients) stratified by PCY sensitivity, using the mean iPCY for their matched clinical treatments as a cutoff. *P* value from log-rank (Mantel–Cox) test and HR of the respective groups including the 95% CIs are reported. Ongoing responses are indicated as vertical tick marks on the Kaplan–Meier curves. Table below reports the number of patients at risk at different time points. **e**, As in **d** but for the immunotherapy subcohort (*n* = 15 patients). **f**, As in **d** but for the non-immunotherapy subcohort (*n* = 19 patients). **g**, As in **e** but stratified for the PGs of the corresponding patient samples. Stratification is PG2 versus (PG1 and PG3). Source data

**Extended Data Fig. 9:**
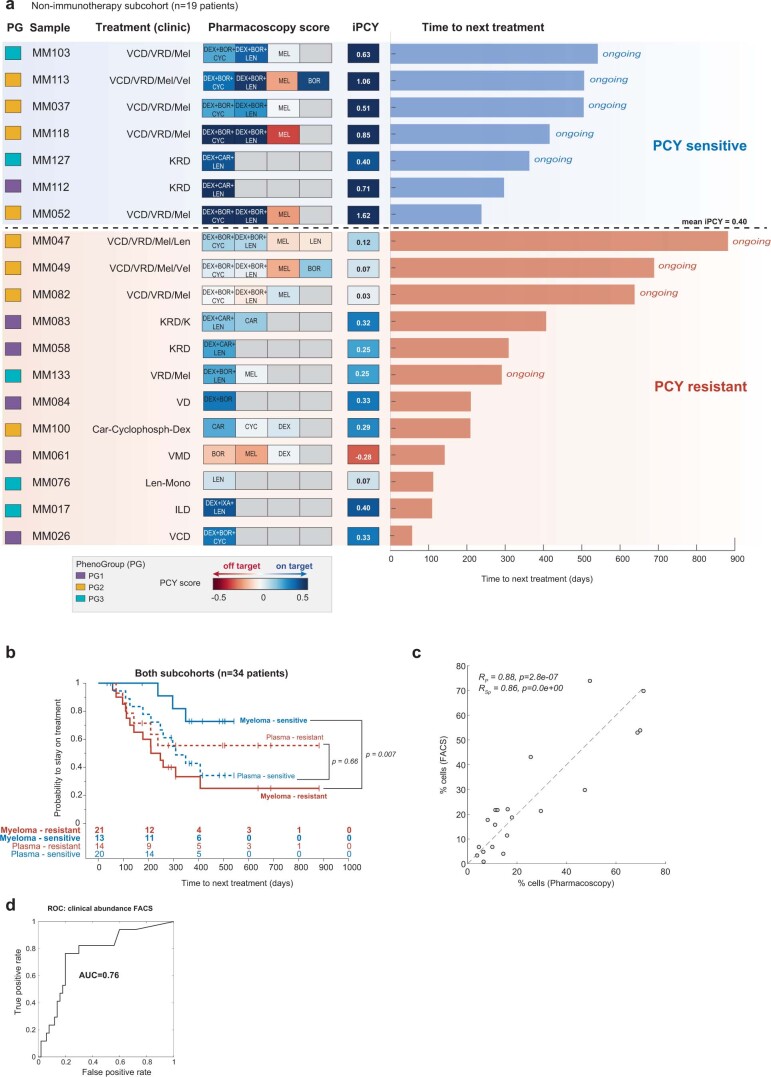
Clinical utility of pharmacoscopy for multiple myeloma. **a**, Graphic representation of the non-immunotherapy subcohort (n = 19 patients), similar to Fig. 8c. Patients with their respective PhenoGroups are reported, followed by the sample IDs, and treatments given in the clinic. A heatmap reports individual PCY scores for the treatments given, with their respective integrated PCY (iPCY) scores on the right. Finally, each patient’s time to the next treatment is reported, with blue indicating PCY-sensitive and red PCY-resistant samples. Ongoing treatments are indicated. **b**, Kaplan-Meier curves showing the time to next treatments in days for all 34 patients (combining both the non-immunotherapy and immunotherapy subcohorts). Thick lines represent cohort stratification by PCY-sensitivity (determined as in Fig. 8b) of myeloma cells. In comparison, dashed lines represent equivalently-calculated cohort stratification by PCY-sensitivity of all plasma cells (as detected by the 4-class CNN classifier). Plasma cell drug responses do not stratify clinical responses. Relevant p-values from the log-rank (Mantel-Cox) test are indicated on the right, with a table reporting the number of patients at risk at different time points below the plot. **c**, Scatter plot showing similarity in subpopulation abundances identified across MM samples by PCY (x-axis) and flow cytometry (y-axis) (n = 5 patient samples across n = 4 subpopulations). Spearman’s rank and Pearson’s correlations and p-values are indicated. **d**, ROC curve on the false (x-axis) and true (y-axis) positive rate of inference of samples belonging to PG1 based on the clinical flow-based abundance of plasma cells in each sample. PG1 inference was performed by thresholding on the plasma cell abundances. Area under the characteristic curve is indicated. ROC curve includes data on 67 patient samples for which the clinical flow data was available. Source data

For a subset of 34 patients in our cohort, the time of sampling and PCY testing preceded the initiation of a new therapy line to which the clinical response (time to next treatment) could be documented (Supplementary Table 8). These clinical response data allowed us to test whether a patient’s ex vivo sensitivity to their next treatment line is predictive of clinical benefit. Given that patients mostly received combination treatments, we summed the PCY scores for all ex vivo tested treatments matching the patient’s next treatment line, resulting in an ‘integrated PCY’ score (iPCY; Fig. 8a). As for the PCY scores, we observed that iPCY scores for patients receiving immunotherapies (‘immunotherapy subcohort’; *n* = 15 patients; Fig. 8b,c) were considerably lower than iPCY scores for patients treatment regimens that did not include immunotherapies (‘non-immunotherapy subcohort’; *n* = 19 patients; Fig. 8b and Extended Data Fig. 9a). We, therefore, labeled patients whose iPCY score was above average of their respective subcohort to be PCY *‘*sensitive’ (Fig. 8b), whereas patients with below average iPCY scores were called ‘resistant’. Kaplan–Meier analysis of all 34 patients showed highly significant increases in the time to next treatment for PCY-sensitive patients compared to PCY-resistant patients (log-rank *P* = 0.00763; hazard ratio (HR) = 4.57, 1.77–11.8 95% confidence interval (CI_95_)) (Fig. 8d).

When analyzing the two subcohorts separately, we found the response to immunotherapy to be significantly stratified by PCY sensitivity before treatment initiation (*P* = 0.0222; HR = 7.01, 1.59–31 CI_95_); Fig. 8e), whereas the non-immunotherapy subcohort trended in the same way but did not reach significance (*P* = 0.0898; HR = 3.49, 1.01–12.1 CI_95_) (Fig. 8f). The phenotypic myeloma signature was further underscored by the observation that repeating the clinical stratification based on the drug responses of the CD138^+^/CD319^+^ plasma cell-marker-positive cells, which is the plasma cell class from the four-class CNN, did not significantly stratify the time to next treatment (Extended Data Fig. 9b). Last, we observed that the clinical responses to immunotherapy were also stratified by the PhenoGroups, with the inflamed PG2 signature indeed associated with shorter time to next treatment, as also predicted by their ex vivo results (*P* = 0.0331; HR = 0.233, 0.0291–1.87 CI_95_) (Figs. 8g and 7c). These results support the here developed ex vivo platform and derived functional precision medicine landscape as a tool to personalize complex immunotherapy-containing treatments for patients with MM.

## Discussion

We here present a single-cell-resolved functional survey of MM, matched to deep molecular and clinical profiling. Our orthogonal datasets are clinically predictive, enable biomarker identification and mechanistic interrogation of myeloma drug sensitivities and might help guide the selection of immunotherapies and combination therapies for precision medicine of MM.

The phenotypic single-cell resolution of the platform is critical, as it is only the ex vivo drug response of the big plasma cell marker-positive cells (the phenotypic myeloma cell signature) that is predictive of clinical response. This phenotype enriches for genetically altered cells, associates with increased disease burden across the cohort and reflects the characteristically high monoclonal antibody production and metabolic activity of myeloma cells. Furthermore, the phenotypic resolution allows in-depth characterization of T cell activation and immune cell engagement with myeloma cells in response to ex vivo immunotherapy treatment.

A recurring thread throughout our findings is the dynamic interplay between the BM environment and the drug sensitivity of myeloma cells, particularly regarding immunotherapy response. The three predominant cell community modes or PGs that we identify by classifying BMNCs across our cohort, are independently supported by orthogonal measurement (imaging, drug response profiling, cytokine profiling, clinical parameters and pathology). These PGs capture the interplay between key myeloma features across our cohort: disease burden and treatment stage, disease clonality and BM inflammation. Jointly, the PGs are the strongest predictors of ex vivo drug sensitivity that we identify. PG1 is characterized by a high cancer burden, high sFLC levels and enriched for newly diagnosed and early-stage MM. PG2 and PG3 enrich for late-stage patients eligible for immunotherapy, yet strongly differ with regards to their ex vivo immunotherapy sensitivity. PG2 is characterized by high monocyte and T cell infiltration into the myeloma niche coinciding with high levels of pro-inflammatory cytokines TNF-α and IL-6. We find that PG2 is significantly associated with poor ex vivo and clinical response to immunotherapies. In contrast, PG3 is high on ‘other’ cells, enriched in later-stage Kappa-clonal patients and shows relatively good ex vivo and clinical response to immunotherapies. Although these patterns are discovered first from real-time drug testing in patient samples, they propose a treatment stratification for patients with later-stage MM that might be reproduced in clinical routine by quantifying BM composition by flow cytometry (Extended Data Fig. 9c,d). Here, of note, ANOVA identified PG2s to be sensitive to the DNA-damaging agent bendamustine (Extended Data Fig. 8a) and PG2 samples in the non-immunotherapy subcohort showed among the best documented clinical responses to standard combinations of bortezomib, dexamethasone and cyclophosphamide (‘VCD’) (Extended Data Fig. 9a), warranting further investigation. Given the strong role of the BM stromal cells (BMSCs) in shaping the microenvironment of MM^11,70^, we expect, but it remains to be shown, that there is an interplay between BMSCs and the identified PGs.

The ex vivo immunotherapy responses measured by PCY significantly stratified clinical response, offering a unique tool to study their molecular and cellular determinants. We find that high expression of the MHC-II molecule HLA-DRB5 on myeloma cells strongly associated with ex vivo elotuzumab response. In the differentiation process from B cells, plasma cells typically lose or lower the expression of MHC-II molecules^71^; however, MHC-II expression on myeloma cells has already been reported^67,72^, hinting that the myeloma cells of this elotuzumab-sensitive subcohort might contain a more plasmablastic phenotype potentially acting as antigen-presenting cells. In line with that interpretation, we observe that patient samples with high myeloma expression of HLA-DRB5 present with increased cell–cell contacts between myeloma cells and activated T cells upon ex vivo elotuzumab treatment. While further work is needed to elucidate this association, it exemplifies the unique ground provided by our dataset for exploring the molecular determinants of immunotherapy response in MM. Going forward, the PCY platform is compatible with bispecific antibodies^50^, immunomodulatory proteins and drugs^51,52^ and CAR-T cell therapy^73^, empowering the further study of recently approved and upcoming myeloma therapies.

Our functional proteomic analysis reveals the molecular network associated with drug sensitivity across treatment options. This comprehensive approach retrieves both well-known associations as well as uncovers new ones. For instance, high abundance in ribosomal proteins, EIF4F translation initiation complex and spliceosomal proteins associated positively with bortezomib sensitivity. This indicates a myeloma cell state engaged in high protein synthesis that is likely sensitive to PI-induced ER stress, aligning well with previous findings^37,74^. DEPTOR, which we identify as top negative association to bortezomib sensitivity, could be an upstream master regulator of this cellular state in myeloma. DEPTOR is an mTOR-interacting protein^63^ that inhibits mTORC1 and mTORC2 pathways that regulate cell growth, proliferation and survival^75^. High DEPTOR expression in MM might thus reduce the overall protein synthesis and associated ER stress, thereby increasing bortezomib resistance. Targeting DEPTOR, possibly in combination with proteasome inhibition, might thus be a promising therapeutic strategy for this subset of patients with DEPTOR-high bortezomib-resistant myeloma.

Finally, our findings support the clinical utility of PCY for personalized treatment identification in MM. Follow-up clinical trials that prospectively assign patients to treatment arms based on their ex vivo drug sensitivity are needed to assess impact on clinical outcome. Altogether, we present a rich resource deeply characterizing MM across disease stages at the phenotypic, functional and molecular level. Our data can be further analyzed for drug target identification, drug mode of action analysis, biomarker detection and patient stratification. It provides a BM classification scheme with potential treatment relevance and supports the future clinical use of our functional precision medicine platform for the treatment of MM.

## Methods

### Obtaining patient bone marrow nuclear cells from iliac crest aspirates

This project was conducted as an observational clinical trial by principles enunciated in the current version of the Declaration of Helsinki and the Swiss regulatory authority’s requirements (BASEC no. 2017-00603, approved by the Cantonal Ethical Committee of Zurich). A comprehensive list of patients enrolled in the study is in Supplementary Table 1. After the patients’ informed consent, BM aspirates were taken, placed in heparin-coated tubes (Beckton Dickenson) and processed within 1 h after aspiration. Samples consisted of approximately 10 ml BM aspirate taken in addition to routine BM biopsy from the iliac crest.

### Drug screening library preparation

After consulting with the MM treating physicians, we obtained a set of US Food and Drug Administration (FDA)-approved compounds and antibodies used in the clinic to design the MM drug-screening library. The drug-screening plates were prepared by direct dilution using acoustic, non-contact transfer with Echo Liquid Handler device Echo (Labcyte). Shortly after, the compounds were transferred to clear-bottom, 384-well tissue-treated plates (PerkinElmer) with three (compounds) or four (antibodies) replicates and three different concentrations. The list of used drugs and concentrations, replicates and abbreviations is detailed in Supplementary Table 5.

### Cell purification, seeding and drug incubation

To isolate BMNCs, 5–10 ml BM aspirate was diluted 1:3 in 2 mM PBS-EDTA (Gibco) and BMNCs were isolated with Histopaque-1077 density gradient (Sigma-Aldrich) according to manufacturer’s instructions. BMNCs at the interface were collected, washed once in PBS and resuspended in RPMI 1640 + GlutaMax medium (Gibco) supplemented with 10% fetal bovine serum (FBS; Gibco). Subsequently, cell number and viability were determined by Countess II Cell Counter (Thermo Fisher) according to the manufacturer’s instructions.

The non-adherent BMNCs were plated at the density of 13,000 cells per well onto a 384-well drug-coated plate. The cells were incubated at 37 °C with 5% CO_2_ for 21–24 h. The assays were stopped by fixing and permeabilizing the cells for 20 min at room temperature (22 °C) with 15 µl fixation solution containing 1% (w/v) formaldehyde (Sigma-Aldrich), 0.1% (v/v) Triton X-100 (Sigma-Aldrich) and PBS. Fixation solution and media were aspired by HydroSpeed plate washer (Tecan) and cells were blocked with 5% FBS–PBS (Gibco) and photobleached with conventional white light LED panels overnight at 4 °C (6–12 h).

### Immunostaining and imaging

All of the antibodies used in this work are listed in Supplementary Table 9. Before immunostaining, the blocking solution was removed and a 20 µl antibody cocktail was added onto the cells. The antibody cocktail for a multiplexed setting consisted of 1:5,000 dilution of DAPI (Sigma-Aldrich) and 1:300 dilution of the following antibodies in PBS: anti-CD138, anti-CD319, anti-CD3 and anti-CD14. The cells were stained for 1 h in the dark at room temperature and then the staining solution was replaced by PBS. For imaging, a PerkinElmer Opera Phenix automated spinning-disk confocal microscope was used. Each well of a 348-well plate was imaged at ×20 magnification with 5 × 5 non-overlapping images, covering the whole-well surface. Imaging was completed in the following, sequential order: brightfield (BF) (650–760 nm), DAPI/nuclear signal (435–480 nm), GFP/green signal (500–550 nm), PE/orange signal (570–630 nm) and APC/red signal (650–760 nm) channel. Subsequently, the raw.tiff images were transferred from the microscope for further analysis. The dataset consists of 62 terabyte of images from over 14 million individual.tiff files.

### Conventional image analysis and quality filtering

Single-cell detection in images was performed using a pipeline in CellProfiler v.2 (ref.^76^) and as previously described by Vladimer et al.^50^. Briefly, to detect cells, nuclei were segmented by thresholding on DAPI intensity, and the cellular outlines were estimated by a circular expansion from the center of the nucleus. Next, a larger expansion outline was added to the nucleus, representing the local cellular area (for example, the staining background of the cell). Finally, CellProfiler features (staining intensity, shape and texture features of the cytoplasm, nucleus and local cell proximity) were extracted for the measured channels.

### Convolutional neural networks

#### Four-class CNN architecture, training and data augmentation

A four-class 71-layer deep CNN with an adapted ResNet architecture^54^ was implemented using MATLAB’s Neural Network Toolbox, as described before^52^. The CNN network was trained on 46,614 individual 48 × 48 × 5 images. The final performance was evaluated on an independent validation dataset of 2,740 images (see below).

#### Four-class CNN: training data generation and normalization

The curated dataset used for the four-class cancer CNN was generated as described previously^52^. In brief, cells used in the CNN training were randomly collected across control (DMSO) and drug-treated conditions. First, indexes of marker-positive cells were obtained, which were assigned to four exclusive classes: plasma cell, T cell, monocyte and other cell. Specifically, a plasma cell was either CD138^+^, CD319^+^ or double-positive (CD138^+^ and CD319^+^). For T cells (CD3^+^), which were multiplexed together with monocytes (CD14^+^) in the red channel, cells from the control single-stained wells were used to determine the intensity and morphology of the subset. The same selection criteria applied to the monocytes. Other cells stained positive for DAPI but were negative for all used subpopulation markers. In total, a dataset of 49,455 individual hand-curated single-cell images across 71 MM patient samples was generated. Before training the CNN, an independent and equally balanced validation dataset was generated containing ten cells per class per patient sample (total of 2,840 individual cells). Overall classification accuracy across the training samples was 95.7%.

#### Big and small plasma cell classifier

To evaluate size differences within the plasma cell population, a manually annotated library of labeled big and small plasma cells across the samples was curated. First, plasma cells were identified by the above-described CNN-based plasma cell identification. Then, plasma cells were randomly selected and cropped into 50 × 50-pixel images around the center of each nucleus containing BF, DAPI and CD138 and CD319-containing fluorescence channels. Finally, cells were manually curated into either ‘big’ or ‘small’ class, based on their nuclear and cytoplasmic size. In total, a training set of 956 big and small cells was curated from across 55 patients with MM. Additionally, a second test set of 200 cells of each subpopulation across all 55 samples was created. A simple eight-layer CNN based on the Alex-net^77^ architecture block design was used. Network performance was assessed by classifying the test cells belonging to each morphological cell class, which were not part of either training or validation datasets. The final B-Net accuracy was 85%.

#### T cell activation classifier

An additional CNN was developed to classify T cells into two morphologies, conventional T cells (T_conv_) and activated T cells (T_act_), as described before^51,52^. Briefly, the T-Net was trained with many non-MM T cells from different experiments, as described before^52^, with the MM T cells in training consisting of 992 T_act_ and 4,769 T_conv_ cells. The T-Net was tested across 265 T_act_ and 593 T_conv_ cells. The cells in the training and testing datasets comprised samples from across 32 patients with MM. The network performance was assessed by classifying the test cells belonging to each morphological T cell class, which were not part of either training or validation datasets. The final T-Net accuracy was 98.6%.

#### Quality control and cellular cleanup

To evaluate cellular images for imaging artifacts, contaminations and correct segmentation, we applied a small custom ‘Cleanup CNN’. This two-class CNN (correct cells and not-correct cells) was based on an architecture known as Alexnet^78^ and was trained as described above for (B-Net). The hand-curated training and validation dataset (10% of the data) consisted of 17,259 individual images, including 25 different patients/experiments randomly sampled across the cohort. After classification of all detected cell objects, all not-correct cells and correct cells with classification confidence lower than 60% were discarded from further analysis.

### Drug response analysis

Measured drug responses were included for 101 MM samples (Supplementary Table 10). Samples not included in this analysis either did not pass the quality control filters (including too low tumor content, microbial contaminations and too high technical variability in drug results) or were initially used for assay setup or eventually used in follow-up experiments.

First, relative cell fractions (RCFs) were calculated as previously described^40^. Briefly, a fraction of cells of population X (as identified by the CNN) after drug treatment divided by the average fraction of population X cells measured in control wells. The antibody-containing treatments were normalized to their respective isotype control. Antibody–drug combinations and other drugs were normalized to solvent DMSO. For calculating PCY ex vivo drug responses, all RCF values per drug were averaged over technical replicates and zero-centered (1–RCF). Therefore, a positive score represents a relative reduction of the respective cell population (on-target effect), whereas a negative score indicates relative ex vivo drug resistance (Supplementary Table 6).

### Quantification of cell–cell contacts (interaction score)

Cell–cell contacts were calculated as described previously^50–52^. For analysis of drug-induced changes in cell–cell contacts, scores were normalized to respective controls, either DMSO or isotype control antibodies.

### Time course measurement of γH2AX expression in myeloma cells

BMNCs of six patients (newly diagnosed or at relapse) (Supplementary Table 10) were isolated as described above and seeded with the density of 13,000 cells per well onto a 384-well plate, coated with DMSO, bortezomib or bendamustine. The cells were incubated at 37 °C with 5% CO_2_ for 1, 6 or 24 h and the incubation was stopped by fixation as described above. The cells were blocked for 1 h using 5 % FBS. Before immunostaining, the blocking solution was removed and a 20 µl antibody cocktail was added onto the cells. The antibody cocktail for consisted of 1:5,000 dilution of DAPI (Sigma-Aldrich) and 1:300 dilution of the following antibodies in PBS: anti-CD138 and anti-phospho-histone H2A.X (Supplementary Table 9). The cells were stained for 1 h in the dark at room temperature and then the staining solution was replaced by PBS. The imaging and subsequent image analysis was conducted as described above. Finally, the percentage of γH2AX-marker-positive myeloma cells was calculated in each drug condition by setting a staining intensity threshold based on its density histograms.

### Cytokine measurements on patient sera

The sera of 48 patient samples were collected after BMNC isolation using a Ficoll density gradient and stored at −20 °C (Supplementary Table 10). Before processing, the selected set of patient sera was thawed and centrifuged at 200*g* for 10 min. The supernatant was collected and frozen until processed by Luminex Cytokine measurement, which was performed by ProtATonce (Greece).

#### Cytokine data analysis

The cytokine measurements were reported as an average cytokine concentration of three technical replicates. If cytokine abundance fell below the limit of detection (as measured by the standard calibration curve), the value was set to not a number (‘NaN’). If the values were above the maximum detectable concentration, they were set to the maximum observed value for that cytokine in the entire cohort. Next, the cytokine concentrations were log_10_-transformed. Then, each cytokine was *z* score normalized across the 48 samples, to account for cytokine abundance differences. Last, the 64 cytokines measured were *z* score normalized per sample to correct for sample input abundance differences (Supplementary Table 2 and Supplementary Table 10).

### Subpopulation isolation

#### MACS sorting

Plasma cells, T cells and monocytes were isolated from fresh BMNCs directly after obtaining them via density centrifugation, as described above. Isolation was performed following Manufacturers’ instructions (Miltenyi Biotec) using a column-based extraction method with CD138, CD3 and CD14 microbeads. Immediately after sorting, the cells were frozen and stored at –80 °C until processed (proteotyping).

#### FACS sorting

FACS sorting was conducted on live BMNCs of five patient samples (Supplementary Table 10), which were isolated using density centrifugation (as described above). Upon isolation, BMNCs were split and washed in a 1:1 ratio in ice-cold FACS buffer (2 mM EDTA, pH 8.0, 0.5% FBS albumin in PBS) by centrifugation (300*g*, 10 min). Next, the cells were stained with antibodies CD138, CD319, CD3 and CD14 (Supplementary Table 9). After 30 min incubation on ice, the cells were washed and resuspended in FACS buffer. The cells were filtered through a 40-μm strainer and stained with viability dye SYTOX Blue (Supplementary Table 9) shortly before commencing sorting.

Single-cell sorting was performed using BD FACSAria Fusion (BD Biosciences). The gating was conducted in the following steps: (1) exclusion of dead cells and debris; (2) selection of the leukocyte population; (3) exclusion of CD14^+^ and CD3^+^ cells; (4) selection of any plasma cell-marker-positive cells (CD138^+^ and/or CD319^+^); and (5) separation by size into ‘big’ and ‘small’ plasma-positive cells using FSC and SSC gates. Cells were analyzed using BD FACSDIVA software (BD Biosciences) and FlowJo software.

Immediately after sorting, the cells were either frozen and stored at –80 °C until processed (proteotyping) or kept in FACS buffer for Cytospin preparation.

### Cytospin preparation and DNA-FISH

Cells from FACS sorting were resuspended to no more than 0.5 × 10^6^ cells per ml in 5% FBS medium. Then, 200 µl suspension was pipetted into the cytofunnel and slides were centrifuged (Thermo Scientific, Cytospin 4) at 450 r.p.m. for 5 min. The slides were removed from the centrifuge and fixed for 15 min at 4 °C in 70% ethanol (Fisher Scientific).

FISH was performed according to the instructions of the manufacturer of the FISH probe (MetaSystems) using the enumeration probe XL 5p15/9q22/15q22 Hyperdiploidy (no. D-5095-100-TC). Then, 100 nuclei were scored for each hybridization.

### Proteotype analysis

#### Sample preparation for mass spectrometry

After MACS or FACS sorting, cells were washed twice with PBS before stored as pellets at −80 °C. Peptides for mass spectrometry measurements were prepared using the PreOmics iST kit (PreOmics). For lysis, the frozen cell pellets were resuspended in a lysis buffer and incubated at 95 °C for 10 min. Subsequently, the samples were sonicated using three 30-s sonication pulses in a VialTweeter (Dr Hielscher). Samples were digested for 2 h at 37 °C and peptides were further purified according to the manufacturer’s protocol.

#### Spectral library generation

For spectral library generation, leftover patient samples were pooled according to their antibody profile (CD3, CD14 and CD138) and fractionated by high pH on an Agilent Infinity 1260 (HP Degasser, Vial Sampler, Cap Pump) and 1290 (Thermostat, FC-μS) system. In short, samples were resuspended in Buffer A (20 mM ammonium formate and 0.1% ammonia solution in water, pH 10) and 100 μg per sample were injected. The peptides were separated on a YMC-Triart C18 reversed-phase column (inner diameter (i.d.) 0.5 mm, length 250 mm, particle size 3 μm and pore size 12 nm) at 30 °C and a flow rate of 8 μl min^−1^. The gradient was a two-step linear gradient with 70 min from 5% to 40% Buffer B (20 mM ammonium formate, 0.1% ammonia solution and 90% acetonitrile in water, pH 10) against Buffer A (20 mM ammonium formate and 0.1% ammonia solution in water, pH 10) followed by 15 min from 40% to 85% Buffer B. The resulting 48 fractions were pooled by column into 12 samples. Samples were analyzed on a Q Exactive HF mass spectrometer (Thermo Fisher Scientific) in data-dependent acquisition mode on an Acclaim PepMap RSLC C_18_, 2 µm, 100 Å, 150 µm i.d. × 150 mm, nanoViper EASY-Spray column (Thermo Fisher Scientific) with the same gradient as for DIA (see below). The mass range was set to m/z 375–1,500 at full MS resolution of 60,000 and an AGC target value of 3 × 10^6^. MS2 scans were recorded at a resolution of 15,000 with an AGC target of 1 × 10^5^. Loop count was set to 10. HCD fragmentation was set to 28 normalized collision energy.

MS/MS spectra assignment was performed with Proteome Discoverer 2.1 (Thermo Fisher Scientific) using Sequest HT and MS Amanda as search nodes together with Percolator. For spectra annotation, a UniProt database filtered for *Homo sapiens* (downloaded April 2018), concatenated with a common contaminant and a standard peptide.fasta file was used. The following search parameters were used for protein identification: (1) peptide mass tolerance set to 10 ppm; (2) MS/MS mass tolerance set to 0.02 Da; (3) fully tryptic peptides with up to two missed cleavages were allowed; (4) carbamidomethylation of cysteine was set as fixed modification; STY phosphorylation, M oxidation, N deamidation and protein N-term acetylation were set as variable modifications. Percolator was set at max delta Cn 0.05, with target FDR strict 0.01 and target FDR relaxed 0.05. Proteome discoverer result files were imported into Spectronaut Pulsar v.12 (Biognosys) for the generation of the spectral library using default parameters.

#### DIA-mass spectrometry measurement and data analysis

Samples were analyzed on a Q Exactive HF mass spectrometer (Thermo Fisher Scientific) equipped with an Easy-nLC 1200 (Thermo Fisher Scientific). Peptides were separated on an Acclaim PepMap RSLC C_18_, 2 µm, 100 Å, 150 µm i.d. × 150 mm, nanoViper EASY-Spray column (Thermo Fisher Scientific). Mobile phase A consisted of HPLC-grade water with 0.1% formic acid and mobile phase B consisted of HPLC-grade ACN (80%) with HPLC-grade water and 0.1% (v/v) formic acid. Peptides were eluted at a flow rate of 1,200 nl min^−1^ using a stepped gradient from 2% to 8% mobile phase B in 4 min, 8% to 32% in 49 min and 32% to 60 % in 1 min.

For DIA, the mass range of m/z 400–1,210 was covered and a full MS was recorded at a resolution of 120,000 with an AGC target value of 3 × 10^6^ and with maximum injection time of 50 ms. The DIA isolation window size was set to 15 m/z and a total of 54 DIA scan windows were recorded at a resolution of 30,000 with an AGC target value of 1 × 10^6^ and a loop count of 18 (ref. ^53^) HCD fragmentation was set to 28 normalized collision and default charge state 3 and with a starting m/z of 200.

DIA data were analyzed using Spectronaut v.14 (Biognosys). MS1 values were used for the quantification process, peptide quantity was set to mean. Data were filtered using Qvalue sparse with a precursor and a protein Qvalue cutoff of 0.01 FDR. Interference correction as well as local cross-run normalization was performed.

Samples with low-input cell numbers were filtered out from the dataset. The data were log_10_-transformed, summarized and the low-abundant proteins were set to NaN. Next, the data was *z* scored across the protein IDs and across the samples. The final dataset consisted of 5,663 unique protein IDs (Supplementary Table 3 and Supplementary Table 10).

### Statistical and computational analysis

#### Statistics and reproducibility

No statistical method was used to predetermine sample size. We excluded samples from analyses whose diagnosis could not be confirmed. Furthermore, we excluded samples that did not meet our quality control standards (too low technical reproducibility or too low cell counts for proteotyping). We provide the detailed list of samples used in the analyses in Supplementary Table 10.

Unless otherwise stated, significance values were calculated with a Student’s *t*-test. Where significance is not shown, it did not reach *P* < 0.05. Spearman’s rank correlation (*R*_Sp_) and Pearson correlation (*R*_P_) are reported for all scatter-plots. For key results, including association analysis of Fig. 7a, *P* values were tested to be consistent between parametric (Student’s *t*-test) and non-parametric (Mann–Whitney *U*-test) significance testing. Multiple testing correction was performed using the FDR procedure introduced by Storey^79^ (implemented under mafdr() function in MATLAB) and is included in the Source Data tables for analysis in Fig. 4 and Fig. 6. In the Source Data (to Fig. 4c; of which Fig. 4d shows individual protein examples) we report both unadjusted and adjusted *P* values for all detected proteins. Unless stated otherwise, data distributions were assumed to be normal, but this was not formally tested. Where applicable, data distributions are shown. Drug screening plate layouts were randomized across the wells of each 384-well plate. Otherwise, no randomization was performed as part of this study. Data collection and analysis were not performed blind to the conditions of the experiments. Further information on research design is available in the Nature Research Reporting Summary linked to this article.

#### CNN latent space feature selection, *t*-distributed stochastic neighbor embedding and spectral clustering

Cells with a CNN class probability >0.6 were chosen at random from the control (DMSO) conditions of samples from 97 patients with MM (Supplementary Table 10). This summed to a total of 489,753 cells, approximating 1,200 cells per sample, equally balanced among the four cell subpopulations.

To choose a representative set of features from the ResNet CNN latent space, PCA of the 512 features across 489,753 cells was performed. The top 100 features with the highest contribution in PC1 and PC2, explaining >85% variability, were chosen for the *t*-SNE representation.

For the set of 489,753 cells depicted in the *t*-SNE embedding, spectral clustering (also called graph clustering) was performed to divide the cells into 15 separate clusters. The remainder of the cells (all the patient cells that were not sampled for *t*-SNE) were *k*-NN-classified into respective spectral clusters. Sample composition, based on the cells in DMSO controls, was calculated and a sample similarity matrix was obtained by correlating cluster abundances per sample. The final PG clustering was robust to changing the arbitrary number of spectral clusters (*k* = 15).

#### Integration of CD138 proteotype data and myeloma abundance by PCY

##### Discovery cohort of 77 patient samples

Upon BMNC isolation, cells were split into two parts. The first was used for PCY, including immunostaining with the original set of surface markers (anti-CD138, anti-CD319, anti-CD3 and anti-CD14), imaging and further analysis by deep learning. This resulted in plasma cell classification into big (myeloma) and small plasma cell-marker-positive morphology types per patient sample. From the second part, plasma cell isolation was performed using MACS separation with anti-CD138 microbeads; these cells were used for proteotyping by DIA-SWATH mass spectrometry. Correlation between protein levels and the abundance of a morphological plasma cell subtype was used to identify putative markers for this subtype. The abundance of each measured protein per sample could be represented and associated with the sample’s respective plasma cell morphology abundance (percentage big and percentage small plasma cells, respectively).

##### Molecular validation cohort of four patient samples

Upon BMNC isolation, cells were immunostained with the same set of surface markers as in the discovery cohort and FACS-sorted based on their maker expression and size into big and small plasma cells. One part of cells was used for single-cell DNA-FISH and the remaining part was analyzed by MS/DIA-SWATH proteotyping and quantification of protein abundances in big and small plasma cells (Supplementary Table 10).

##### Obtaining molecular signature of myeloma cells

To obtain a molecular signature of myeloma cells: (1) The molecular proteotype of myeloma cells was computationally inferred by correlation of fraction of big cells among plasma cells and plasma cell proteotypes. Spearman correlation coefficient was used and the correlation *P* values were taken to calculate the signed log_10_(*P*) values; (2) The fold change in protein abundance between FACS-sorted big and small plasma cells from four samples was calculated. *P* values were calculated using a paired *t*-test; (3) Fisher’s exact test was used to calculate significance in reproducibility of proteins, significant in both discovery and validation cohorts (*P* < 5.47 × 10^−5^); (4) To select a molecular signature of myeloma cells, a significance cutoff for the validation cohort was set to *P* < 0.05 and for the validation cohort, an absolute difference in log_2_(fold change) > 0.3.

The molecular signature was further used in the scRNA-seq analysis (see below). The list of signature proteins is provided in Supplementary Table 4.

#### Analysis of scRNA-seq dataset

For Fig. 4, scRNA-seq count matrices and associated metadata were obtained from the Gene Expression Omnibus (accession code GSE161195) on 5 November 2020. The scRNA-seq dataset was from Cohen et al.^39^ Raw count matrices were first filtered to remove cells with fewer than 300 unique molecular identifiers (UMIs) (not counting UMIs originating from immunoglobulin genes) or more than 50% mitochondrial gene content. Next, cells that were likely not plasma cells were identified by calculating a cell type score using the SingScore method (v.1.12.0)^80^ and the following gene sets from the molecular signatures database (msigdbr v.7.4.1):

‘CUI_DEVELOPING_HEART_C3_FIBROBLAST_LIKE_CELL’ ‘HAY_BONE_MARROW_CD8_T_CELL’

‘HAY_BONE_MARROW_IMMATURE_NEUTROPHIL’

‘HAY_BONE_MARROW_MONOCYTE’

‘HAY_BONE_MARROW_NAIVE_T_CELL’

‘HAY_BONE_MARROW_NEUTROPHIL’

‘HAY_BONE_MARROW_NK_CELLS’

‘HAY_BONE_MARROW_PLASMA_CELL’

‘HAY_BONE_MARROW_PLATELET’

‘HAY_BONE_MARROW_STROMAL’

Cells with low plasma cell scores, but high scores for other cell types as well as any cell expressing *S100A8*, *CD14*, *CD3D*, *CD3E*, *TRAC*, *COL1A2* or *C1QA* were excluded.

In addition, the dataset was subset to include only relapsed and refractory MM samples (sample_characteristics_ch1.6, disease_state, PRMM) at baseline (sample_characteristics_ch1.4, time point, baseline). Overall, 31,305 cells from 34 patient samples were retained.

The gene expression was subset to use only genes matching the protein IDs with the myeloma and small plasma cell signature, determined as described above (also, see Supplementary Table 4). Genes not detected in any cell as well as immunoglobulin genes were omitted from analysis.

Next, each cell was assigned a score indicating similarity to big plasma cells in our proteomics dataset. This was calculated as the mean expression of big associated genes divided by the mean expression of small associated genes (Supplementary Table 4). A cell was assigned to belong to a big-like phenotype if its score was higher than the median of all scores.

The fraction of big-like cells per patient was calculated and associated with the disease state (sample_characteristics_ch1.7, cohort, refractory or cohort, relapse) using a two-tailed Student’s *t*-test.

#### LASSO regression for inference of missing drug responses

Strictly for Fig. 5 visualization purposes, LASSO regression was used to predict (infer) myeloma PCY drug responses that were not experimentally measured. Predictions were made either based on measured myeloma drug responses or on matched proteotype data. Linear LASSO regression was run including a 100-parameter Lambda scan to minimize the deviance in a tenfold cross validation setting, performed using the lassoglm() implementation (MATLAB). The model with the best performance was chosen and only models with a 0.8 or higher Pearson’s (linear) correlation between predicted and measured (training) values were kept and used for inference.

#### Elastic net regression for identification of protein predictors of drug response

Elastic net regression was used to predict (infer) myeloma PCY drug responses and top-three positive and negative predictive coefficients are listed in Supplementary Table 7. Elastic net regression was run including a 100-parameter Lambda scan to minimize the deviance in a tenfold cross validation setting, performed using the lassoglm() implementation (MATLAB), with the α parameter set to 0.5. Coefficients are reported for the model corresponding to the Lambda value that minimizes the deviance.

#### Protein–drug associations and STRING-db interaction network

For the molecular analyses (Fig. 6), the effect of differential myeloma content in the plasma cell samples (MACS-isolated by CD138) was regressed out and the residual protein expression was used. Ex vivo drug responses were then correlated (Spearman’s rank correlation) with the residual protein expression (data that were integrated in Supplementary Tables 3 and 6 and samples used in Supplementary Table 10).

The STRING-db interaction network for the proteins whose abundance in myeloma cells most strongly correlated with myeloma drug responses across all proteins and drugs was used for network representation. Proteins were selected for having an absolute (*R*_sp_) > 0.585 with *P* < 0.05 (thresholds chosen to generate a reasonable network size), as well as the top-two and bottom-two strongest associations per tested drug or drug combination. Unconnected nodes in the network were omitted from the visualization for simplicity. Node color was determined by the Spearman’s rank correlation of a drug response across the discovery cohort.

#### Multiple myeloma cell line analysis through DepMap

For Fig. 6, publicly accessible drug response and transcriptomics data from myeloma cell lines were obtained through the DepMap portal (https://www.depmap.org/portal). Cell lines were filtered for being plasma or myeloma-derived cell lines analyzed previously^66^, annotated as ‘Sanger GDSC1’ in DepMap, in the form of natural log-transformed half-maximum inhibitory concentration values in micromolar. Transcriptomics data were log_2_-transformed(1 + transcript per million) and annotated in DepMap as ‘Expression 22Q1 Public’.

#### One-way ANOVA on drug responses for different features

One-way ANOVA was performed (Fig. 7) to evaluate the influence of different clinical and morphological features on the drug responses. Only patients with active, clonal disease were selected (*n* = 67) for the analysis and *n* values differed depending on clinical feature availability (Supplementary Table 10). The ANOVA *F* values and the Student’s *t*-test values were used for representing the features associated with drug responses in the network, with the *F* and *P* values from both tests filtered at significance of 0.01 (Supplementary Table 5).

#### Survival analysis

For both subcohorts combined (*n* = 34 patients), patients were stratified by sensitivity, using the mean iPCY for their matched clinical treatments as cutoff. For immunotherapy subcohort (*n* = 15 patients), mean sensitivity of iPCY of 0.21 was used; for the non-immunotherapy (*n* = 19 patients), iPCY of 0.40 was used as a cutoff. *P* values from log-rank (Mantel–Cox) test and HR of the respective groups including the 95% CIs were calculated and Kaplan–Meier curves for the probability to stay on treatment were calculated using the MatSurv function in MATLAB. Patient treatment regimes are summarized in Supplementary Table 8.

### Reporting summary

Further information on research design is available in the Nature Portfolio Reporting Summary linked to this article.

## Supplementary information

Reporting Summary

Supplementary Tables Supplementary Tables 1–10. Table titles and legends are contained within the file.

## Data Availability

The data are available as supplementary and source data tables and are accessible and interrogatable at https://myelomics.com. Mass spectrometry raw files have been deposited to MassIVE (https://massive.ucsd.edu/) with dataset identifier MSV000088992, available also at 10.25345/C58S4JS3T. Previously published scRNA-seq data from the KYDAR study that were re-analyzed here are available under accession code GSE161195 (ref. ^39^). Publicly accessible drug response and transcriptomics data from myeloma cell lines were obtained through the DepMap portal (https://www.depmap.org/portal). Additional databases used in the study include: UniProt (https://uniprot.org, release 2018_1), STRING-db (https://string-db.org, v.11.5) and the Molecular Signatures Database (https://www.gsea-msigdb.org/gsea/msigdb/, v.2021.1.Hs). All other data supporting the findings of this study are available from the corresponding author on reasonable request. Source data are provided with this paper.

## Source data

Source Data Fig. 2 Statistical Source Data for Fig. 2.
Source Data Fig. 3 Statistical Source Data for Fig. 3.
Source Data Fig. 4 Statistical Source Data for Fig. 4.
Source Data Fig. 5 Statistical Source Data for Fig. 5.
Source Data Fig. 6 Statistical Source Data for Fig. 6.
Source Data Fig. 7 Statistical Source Data for Fig. 7.
Source Data Fig. 8 Statistical Source Data for Fig. 8.
Source Data Extended Data Fig. 1 Statistical Source Data for Extended Data Fig. 1.
Source Data Extended Data Fig. 2 Statistical Source Data for Extended Data Fig. 2.
Source Data Extended Data Fig. 3 Statistical Source Data for Extended Data Fig. 3.
Source Data Extended Data Fig. 4 Statistical Source Data for Extended Data Fig. 4.
Source Data Extended Data Fig. 5 Statistical Source Data for Extended Data Fig. 5.
Source Data Extended Data Fig. 6 Statistical Source Data for Extended Data Fig. 6.
Source Data Extended Data Fig. 7 Statistical Source Data for Extended Data Fig. 7.
Source Data Extended Data Fig. 8 Statistical Source Data for Extended Data Fig. 8.
Source Data Extended Data Fig. 9 Statistical Source Data for Extended Data Fig. 9.

## Code availability

Image analysis was performed using the open source CellProfiler package available at https://www.cellprofiler.org. All other analyses were performed using standard MATLAB (R2020a and R2020b) and R code.
