# Supplementary material for: Ex vivo drug response heterogeneity reveals personalized therapeutic strategies for patients with multiple myeloma
Source: Nat Cancer. 2023 Apr 20;4(5):734–53. doi: 10.1038/s43018-023-00544-9 (PMC10212768; doi:10.1038/s43018-023-00544-9)
Supplement: Supplementary file 1 — Reporting Summary [file 43018_2023_544_MOESM1_ESM.pdf]

nature portfolio

Corresponding author(s): Berend Snijder

Last updated by author(s): 03.03.2023

Reporting Summary

Nature Portfolio wishes to improve the reproducibility of the work that we publish. This form provides structure for consistency and transparency in reporting. For further information on Nature Portfolio policies, see our Editorial Policies and the Editorial Policy Checklist.

Statistics

For all statistical analyses, confirm that the following items are present in the figure legend, table legend, main text, or Methods section.

n/a	Confirmed	
☐	☒	The exact sample size (*n*) for each experimental group/condition, given as a discrete number and unit of measurement
☐	☒	A statement on whether measurements were taken from distinct samples or whether the same sample was measured repeatedly
☐	☒	The statistical test(s) used AND whether they are one- or two-sided<br>*Only common tests should be described solely by name; describe more complex techniques in the Methods section.*
☐	☒	A description of all covariates tested
☐	☒	A description of any assumptions or corrections, such as tests of normality and adjustment for multiple comparisons
☐	☒	A full description of the statistical parameters including central tendency (e.g. means) or other basic estimates (e.g. regression coefficient) AND variation (e.g. standard deviation) or associated estimates of uncertainty (e.g. confidence intervals)
☐	☒	For null hypothesis testing, the test statistic (e.g. *F*, *t*, *r*) with confidence intervals, effect sizes, degrees of freedom and *P* value noted<br>*Give P values as exact values whenever suitable.*
☐	☐	For Bayesian analysis, information on the choice of priors and Markov chain Monte Carlo settings
☐	☐	For hierarchical and complex designs, identification of the appropriate level for tests and full reporting of outcomes
☐	☒	Estimates of effect sizes (e.g. Cohen's *d*, Pearson's *r*), indicating how they were calculated

Our web collection on statistics for biologists contains articles on many of the points above.

Software and code

Policy information about availability of computer code

Data collection	All data was collected with commercially available and/or previously published computational methods. See Materials and Methods section for further details.
Data analysis	Data analysis we predominantly performed using Matlab versions R2020a and R2020b. Indidiual data types have been analyzed using commercially available and/or previously published code as described in the Materials and Methods section.<br><br>FACSDIVA software (BD Biosciences), version 6.1.2<br>FlowJo software (BD Biosciences), version 10<br>Matlab (MathWorks), versions 2020a and 2020b<br>R (open source), version 4.1.3<br>CellProfiler (open source; Broad Institute), version 2<br>Spectronaut Pulsar (Biognosys), v12<br>SingScore method (version 1.12.0)

For manuscripts utilizing custom algorithms or software that are central to the research but not yet described in published literature, software must be made available to editors and reviewers. We strongly encourage code deposition in a community repository (e.g. GitHub). See the Nature Portfolio guidelines for submitting code & software for further information.

Data

Policy information about availability of data

All manuscripts must include a data availability statement. This statement should provide the following information, where applicable:

- Accession codes, unique identifiers, or web links for publicly available datasets
- A description of any restrictions on data availability
- For clinical datasets or third party data, please ensure that the statement adheres to our policy

The data are available as supplementary and source data tables, and are further accessible and interrogatable at https://myelomics.com.

Mass spectrometry raw files have been deposited to MassIVE (https://massive.ucsd.edu/) with dataset identifier MSV000088992, available also at https://doi.org/doi:10.25345/C58S4JS3T.

Previously published scRNAseq data from the KYDAR study that were re-analysed here are available under accession code GSE16119539.

Publicly accessible drug response and transcriptomics data from myeloma cell lines were obtained through the DepMap portal (https://www.depmap.org/portal).
OOther databases used in this study:
UniProt, https://uniprot.org, Release 2018_1
String Database, https://string-db.org, version 11.5
Molecular Signatures Database (MSigDB), https://www.gsea-msigdb.org/gsea/msigdb/, v2021.1.Hs

All other data supporting the findings of this study are available from the corresponding author on reasonable request.

Human research participants

Policy information about studies involving human research participants and Sex and Gender in Research.

Reporting on sex and gender	Gender information was not considered or collected as part of this study. Sex information was not considered as part of the study design. Sex information was collected as part of this study, based on participants self reporting. Sex information on each sample donor in the cohort is available as part of Supplementary Table 1. Source Data Tables with data per sample report the sample id, allowing to re-stratify results based on the sex of the sample donor. The cohort includes 44 samples from female patients, and 94 samples from male patients. This sex imbalance is reflective of the increased prevalence of multiple myeloma among men.
Population characteristics	Please see Supplementary Table 1 for the full details per individual sample and patient. Our cohort includes 138 samples from 99 unique multiple myeloma patients. Their average age is 63 years old. 44 samples came from female patients. 94 samples came from male patients. This sex and age bias is representative of the disease incidence of multiple myeloma, which preferentially affects older men. All additionally collected population characteristics are provided in Supplementary Table 1.
Recruitment	Patients over 18 years entering the University Hospital Zurich during routine procedure with confirmed multiple myeloma were eligible and invited to enroll in the study. No explicit effort was performed to avoid further recruitment bias, leading to a sex and age bias that is inherent to the prevalence of multiple myeloma.
Ethics oversight	Ethics oversight was performed by the Kantonale Ethikkommission from the Kanton Zurich (https://www.zh.ch/de/gesundheitsdirektion/ethikkommission.html). The study received ethical approval, with BASEC-number 2017-00603.

Note that full information on the approval of the study protocol must also be provided in the manuscript.

Field-specific reporting

Please select the one below that is the best fit for your research. If you are not sure, read the appropriate sections before making your selection.

☒ Life sciences ☐ Behavioural & social sciences ☐ Ecological, evolutionary & environmental sciences

For a reference copy of the document with all sections, see nature.com/documents/nr-reporting-summary-flat.pdf

Life sciences study design

All studies must disclose on these points even when the disclosure is negative.

Sample size	No sample size calculation was performed to predetermine appropriate sample sizes. Our sample sizes build on succesful previous studies, for example https://www.nature.com/articles/s41591-021-01232-w

Data exclusions	9 samples were initially excluded due to wrong diagnosis at the time of sampling
Replication	Replication of the study measurements occured at different levels: At the highest level, measurements have been replicated across independent patient samples (e.g. Figures 1b, 2e-h, 3a-e, 4d,g,h, 5, 6c,d,f,g,j,k, 7b,c, 8b-g). Therefore, all replication results are reported in the figures and corresponding source data tables.
Randomization	Prospective observational clinical studies are not interventional and therefore not randomized. Randomization of drugs and drug combinations on 384-well drug screening plates did occur.
Blinding	No blinding was performed as part of this study. However, as this was a prospective observational study, the clinical drug response and outcomes were unknown to the researchers at the time of performing the ex vivo image-based drug response (pharmacoscopy) measurements. Clinical drug responses typically were only documented several months after the experiments were completed.

Reporting for specific materials, systems and methods

We require information from authors about some types of materials, experimental systems and methods used in many studies. Here, indicate whether each material, system or method listed is relevant to your study. If you are not sure if a list item applies to your research, read the appropriate section before selecting a response.

Materials & experimental systems

n/a	Involved in the study
☐	☒ Antibodies
☒	☐ Eukaryotic cell lines
☒	☐ Palaeontology and archaeology
☒	☐ Animals and other organisms
☐	☒ Clinical data
☒	☐ Dual use research of concern

Methods

n/a	Involved in the study
☒	☐ ChIP-seq
☐	☒ Flow cytometry
☒	☐ MRI-based neuroimaging

Antibodies

Antibodies used	Anti-human CD3, Alexa Fluor 647, UCHTl, (used in multiplexed), BioLegend, Cat# 300416, Lot# B246715<br>Anti-human CD14, Alexa Fluor 647, HCD14, (used in multiplexed), BioLegend, Cat# 325612, Lot# B244280<br>Anti-human CD138 (Syndecan-1), FITC, DL-101, lnvitrogen, Cat# 11-1389-42, Lot# 2124617<br>Anti-human CD319 (CRACC), PE, 162.1, BioLegend, Cat# 331806, Lot# B268818<br>Anti-human Phospho-Histone H2A.X(Ser139), PE, 20E3, Cell Signaling Technology, Cat# 5763, Lot# 8
Validation	The anti-human CD3 antibody is quality control tested by the manufacturer using immunofluorescent staining with flow cytometric analysis of human peripheral blood lymphocytes, and further verified using fluorescent immunocytochemistry on human peripheral mononuclear cells and and immunohistochemistry on frozen human tonsil tissue.<br>The anti-human CD14 antibody is quality control tested by the manufacturer using immunofluorescent staining with flow cytometric analysis of human peripheral blood monocytes, and further verified using fluorescent immunocytochemistry on human peripheral mononuclear cells.<br>The anti-human CD138 antibody is quality control tested by the manufacturer using immunofluorescent staining with flow cytometric analysis of U266 cells.<br>The anti-human CD319 antibody is quality control tested by the manufacturer using immunofluorescent staining with flow cytometric analysis of human peripheral blood lymphocytes.<br>The anti-human Phospho-Histone H2A.X(Ser139) antibody is quality control tested by the manufacturer using immunofluorescent staining with flow cytometric analysis of HT-29 cells untreated and treated with UV (100mJ/cm2 with 2hr recovery).

Clinical data

Policy information about clinical studies
All manuscripts should comply with the ICMJE guidelines for publication of clinical research and a completed CONSORT checklist must be included with all submissions.

Clinical trial registration	Our observational study was not pre-registered. Our ethical approval reports online on our study: BASEC-number 2017-00603 (see https://ongoingprojects.swissethics.ch/runningProjects_list.php?q=%28BASECID~contains~2017-00603%29&orderby=dBASECID)
Study protocol	Study protocol of this observational study is not available online, but was made available in print to all patients prior to enrollment.
Data collection	All samples and clinical data were collected at the University Hospital Zurich. Sampling dates range from 13.07.2017 until 29.06.2021, with the last clinical update documented on 17.10.2021.
Outcomes	Primary and secondary endpoints of this observational study were predefined in the study protocol, including Primary: Feasibility of pharmacoscopy testing on MM samples, and Secondary: Statistical association between ex vivo drug response and clinical response defined as time to next treatment, analyzed by KM analysis.

Flow Cytometry

Plots

Confirm that:

☒ The axis labels state the marker and fluorochrome used (e.g. CD4-FITC).

☒ The axis scales are clearly visible. Include numbers along axes only for bottom left plot of group (a 'group' is an analysis of identical markers).

☒ All plots are contour plots with outliers or pseudocolor plots.

☒ A numerical value for number of cells or percentage (with statistics) is provided.

Methodology

Sample preparation	Upon isolation, BMNCs were split and washed in a 1:1 ratio in ice-cold FACS buffer (2 mM EDTA, pH 8.0, 0.5% foetal bovine serum albumin in PBS) by centrifugation (300g, 10 min). Next, the cells were stained with antibodies CD138, CD319, CD3, and CD14 (Supplementary Table S8). Upon 30 min incubation on ice, the cells were washed and resuspended in FACS buffer. The cells were filtered through a 40-µm strainer and stained with viability dye SYTOX Blue (Supplementary Table S8) shortly before commencing sort.
Instrument	BD FACSAria Fusion (BD Biosciences)
Software	BD FACSDIVA software (BD Biosciences) and FlowJo software
Cell population abundance	Sorting at least 20.000 single cells for downstream analysis (FISH and proteotyping).
Gating strategy	First, FSC-A and SSC-A gates are used to select the lymphocytes that are further enriched in viable cells. CD14 and CD3 cells are excluded, and only plasma cells (CD138 and/or CD319 positive) are kept. Finally, plasma cells are separated based on SSC-A and FSC-A gates into big (myeloma) and small plasma-marker positive cells. Singlets of each subpopulation are chosen and sorted out for further downstream processing.

☒ Tick this box to confirm that a figure exemplifying the gating strategy is provided in the Supplementary Information.
